# Single-Cell RNA-Sequencing and Metabolomics Analyses Reveal the Contribution of Perivascular Adipose Tissue Stem Cells to Vascular Remodeling

**DOI:** 10.1161/ATVBAHA.119.312732

**Published:** 2019-07-25

**Authors:** Wenduo Gu, Witold N. Nowak, Yao Xie, Alexandra Le Bras, Yanhua Hu, Jiacheng Deng, Shirin Issa Bhaloo, Yao Lu, Hong Yuan, Efthymios Fidanis, Alka Saxena, Tokuwa Kanno, A. James Mason, Jozef Dulak, Jingjing Cai, Qingbo Xu

**Affiliations:** 1 From the School of Cardiovascular Medicine and Sciences, King’s College London, BHF Centre, United Kingdom (W.G., W.N.N., Y.X., A.L.B., Y.H., J. Deng, S.I.B., Q.X.); 2 Center of Clinical Pharmacology, Department of Cardiology, Third Xiangya Hospital, Central South University, Changsha, China (Y.L., H.Y., J.C.); 3 Genomics Research Platform, Biomedical Research Centre at Guy’s Hospital, London, United Kingdom (E.F., A.S.); 4 Institute of Pharmaceutical Science, School of Cancer & Pharmaceutical Science, King’s College London, Franklin-Wilkins Building, London, United Kingdom (T.K., A.J.M.); 5 Department of Medical Biotechnology, Faculty of Biochemistry, Biophysics and Biotechnology, Jagiellonian University, Kraków, Poland (J. Dulak).

**Keywords:** adipose tissue, homeostasis, regeneration, stem cells, vascular remodeling

## Abstract

Perivascular adipose tissue (PVAT) plays a vital role in maintaining vascular homeostasis. However, most studies ascribed the function of PVAT in vascular remodeling to adipokines secreted by the perivascular adipocytes. Whether mesenchymal stem cells exist in PVAT and play a role in vascular regeneration remain unknown.

**Approach and Results:**

Single-cell RNA-sequencing allowed direct visualization of the heterogeneous PVAT-derived mesenchymal stem cells (PV-ADSCs) at a high resolution and revealed 2 distinct subpopulations, among which one featured signaling pathways crucial for smooth muscle differentiation. Pseudotime analysis of cultured PV-ADSCs unraveled their smooth muscle differentiation trajectory. Transplantation of cultured PV-ADSCs in mouse vein graft model suggested the contribution of PV-ADSCs to vascular remodeling through smooth muscle differentiation. Mechanistically, treatment with TGF-β1 (transforming growth factor β1) and transfection of microRNA (miR)-378a-3p mimics induced a similar metabolic reprogramming of PV-ADSCs, including upregulated mitochondrial potential and altered lipid levels, such as increased cholesterol and promoted smooth muscle differentiation.

**Conclusions:**

Single-cell RNA-sequencing allows direct visualization of PV-ADSC heterogeneity at a single-cell level and uncovers 2 subpopulations with distinct signature genes and signaling pathways. The function of PVAT in vascular regeneration is partly attributed to PV-ADSCs and their differentiation towards smooth muscle lineage. Mechanistic study presents miR-378a-3p which is a potent regulator of metabolic reprogramming as a potential therapeutic target for vascular regeneration.

## Abstract 2

Supplemental Digital Content is available in the text.

**Highlights**

- Single-cell RNA-sequencing allows direct visualization of perivascular adipose tissue-derived mesenchymal stem cell heterogeneity at a single-cell level and uncovers 2 subpopulations with distinct signature genes and signaling pathways.
- The function of perivascular adipose tissue in vascular regeneration is partly attributed to perivascular adipose tissue-derived mesenchymal stem cells and their differentiation towards smooth muscle lineage.
- TGF (transforming growth factor)-β1 and miR-378a-3p mimics induced a similar metabolic reprogramming of perivascular adipose tissue-derived mesenchymal stem cells, including upregulated mitochondrial potential and altered lipid levels such as increased cholesterol and promoted smooth muscle differentiation.

Perivascular adipose tissue (PVAT) anatomically abuts the adventitial side of the artery.^1^ Changes in the phenotype of PVAT correlates with disease progression, such as atherosclerosis, and the increase of volume and upregulation of inflammatory factors secreted by adipocytes are associated with worse outcomes.^2^ Loss of PVAT resulting from the specific deletion of PPAR-γ (peroxisome proliferator-activated receptor γ) in murine smooth muscle cells (SMCs) contributes to endothelial dysfunction and impairs vascular homeostasis.^3^ Moreover, transplantation of PVAT to the adventitial side of femoral artery after de-endothelialization injury accelerates neointimal hyperplasia.^4^ However, most studies have attributed the function of PVAT to cytokines such as MCP-1 (monocyte chemoattractant protein 1), and little is known about the possible impact of stem cells in the PVAT surrounding the vascular wall.^3,4^

**See accompanying editorial on page 1896**

Tissue-resident stem cells, including vascular progenitors, are responsible for tissue remodeling.^5,6^ Adventitial stem/progenitor cells participate in vascular remodeling through migration towards the injury site and differentiation towards vascular lineages including SMCs.^7^ Adipose tissue-derived mesenchymal stem/stromal cells (ADSCs) from the subcutaneous and visceral fat display the potential to differentiate towards vascular lineages, including endothelial cells (ECs) and SMCs.^8,9^ Therefore, it remains to be explored whether PVAT-derived mesenchymal stem cells (PV-ADSCs) exist in the PVAT and contribute to vascular remodeling through vascular lineage differentiation in vivo like adventitial stem/progenitor cells.

Mesenchymal stem cells (MSCs) from different tissues origins display similar phenotypic characteristics and multilineage differentiation potential in vitro, however, their well-documented heterogeneity has hindered major research progress in the field. Single-cell RNA-sequencing (scRNA-seq) analysis allows transcriptome profiling at the single-cell level and might help to gain further insight about distinctive subpopulations.^10^ Recent studies have shown that metabolism which primarily sustains the energy need of stem cells regulates pluripotency and differentiation.^11^ For example, fatty acid oxidation was found to be important for hematopoietic stem cell maintenance.^12^ To date, whether the induction of SMC differentiation by TGF-β1 (transforming growth factor β1) is, in part, due to the metabolic regulation has not been investigated.

In this study, we demonstrate the heterogeneity of PV-ADSCs at a single-cell level and uncover 2 distinct clusters with specific signature markers and signaling pathways. Next, we show that PV-ADSCs participate in vascular remodeling in vivo notably through SMC differentiation. In addition, we demonstrate that during the SMC differentiation of PV-ADSCs, TGF-β1 and microRNA (miR)-378a-3p induce a metabolic reprogramming consisting of increased mitochondrial oxidative metabolism, upregulated mitochondrial potential, and altered lipid levels, such as increased cholesterol level. Therefore, our study highlights the heterogeneity of PV-ADSCs at the single-cell level, unravels their importance in vascular remodeling and presents miR-378a-3p as a potential therapeutic target to prevent pathological vascular remodeling.

## Materials and Methods

The data that support the findings of this study are available from the corresponding author upon reasonable request. Detailed materials and methods are in the online-only Data Supplement.

### Experimental Mice

All animal procedures were approved by the UK Home Office. C57BL/6J mice were purchased from The Jackson Laboratory. Male mice aged 8 to 10 weeks were selected for surgery. Only male mice were selected for minimization of data variation brought by difference of sex. Breeding pairs of miR-378a-knockout mice (129SvEv/C57BL/6/mir-378-KO) were kindly provided by Dr Eric Olson (University Texas Southwestern Medical Centre, Dallas, TX) and crossed with C57Bl/10ScSn-Dmdmdx/J mice for testing miR-378a effects in Duchenne muscular dystrophy (other work, in preparation). Protocols from the Institutional Committee for Use and Care of Laboratory Animal and license issued by Home Office, United Kingdom, were followed in all animal procedures.

### Primary Culture of Adipose Tissue-derived Stem Cells from Mouse

Periaorta adipose tissues surrounding thoracic aorta (from the aortic arch to the aortic hiatus) of five 8-week-old C57BL/6J mice were pooled for each primary culture. Finely minced adipose tissue was washed with PBS once and then digested with 2 mg/mL collagenase type I (Life Tech, 17018–029) for 30 minutes at 37°C in a shaker with a speed set at 100 rpm. The pellet was resuspended in stem cell culture medium (α- minimal essential medium [MEM] with 15% embryomax [Millipore, ES-009-B], 0.1 mmol/L 2-mercaptoethanol [Sigma], 10 ng/mL recombinant human leukemia inhibitory factor [Chemicon, Temecula, CA], 5 ng/mL bFGF [basic fibroblast growth factor; R&D systems], 2 mmol/L L-glutamine [Sigma], 100 U/mL penicillin, and 100 mg/mL streptomycin [GIBCO, Grand Island, NY]) and placed in a 5% Co_2_ incubator. ADSCs from miR-378a knockout mice were isolated from the inguinal subcutaneous adipose tissue and cultured in the same manner. Cells within 10 passages were used.

### Phenotyping of Primary and Cultured PV-ADSC

Primary cells obtained after the enzymatic digestion of periaorta adipose tissue or cultured cells detached with scraptase (GenDEPOT) were stained for 30 minutes at 4°C with following antibodies: anti-CD45-APC (BD Biosciences, 561018), anti-CD29-PE (BD Biosciences, 562801), anti-Sca-1 (stem cell antigen 1)-PE-Cy7 (Biolegend, 108113), anti-CD31-PerCP (Biolegend, 201419), anti-CDH5-Alexa Fluor 647 (BD Biosciences, 562242), anti-PDGFRα-APC (platelet-derived growth factor α; eBioscience, 17-1401-81), anti-CD117-PE (Biolegend, 105807), anti-CD34-APC (Biolegend, 128611), anti-CD44-PerCP (Biolegend, 103035), and anti-CD11b-PE (Biolegend, 101205). Nucleated cells were distinguished from debris with Syto16 (Molecular Probes, S7578) and dead cells with 4′,6-diamidino-2-phenylindole (DAPI). Cells were analyzed with BD Accuri C6 or BD LSR Fortessa II (both Becton Dickinson). Gating was set with appropriate fluorescence minus one controls or corresponding IgG controls.

### Sorting and scRNA-Seq of PV-ADSCs

Periaorta adipose tissue surrounding thoracic aorta (from the aortic arch to the aortic hiatus) was enzymatically digested to obtain primary PV-ADSCs. The cell suspensions were stained with Syto16, DAPI, CD45, CDH5, CD29, and Sca1 as described earlier in the phenotyping of primary and cultured PV-ADSCs. After proper gating, the single Syto16^+^/DAPI^−^/CD45^−^/CDH5^−^/CD29^+^/Sca1^+^ population were taken for subsequent scRNA-seq. RNA libraries were prepared on the contactless liquid handling system Labcyte Echo 525 (Labcyte Inc). Data quality was assessed with FastQC, reads were aligned to mouse genome (mm^9^) with HiSat2 2.0.5, sorted with SamTools 1.4 and assembled with StringTie 1.3.3.^13^ R package scater was used for quality control (85 out of 94 cells with aligned reads >800 000 were selected for further analysis) and normalization (default scaling normalization).^14^ Automated Single-cell Analysis Pipeline with log-transformed and normalized values as input was used for multidimensional scaling, clustering, differential expression (SCDE).^15^ Heatmaps and violoin plots of scRNA-seq data were plotted with R package ggplot2. Gene ontology and Kyoto encyclopedia of genes and genomes pathway analysis were performed with DAVID website.^16^ Gene set enrichment analysis was performed with GSEA software.^17^

For scRNA-seq of cultured PV-ADSCs, enzymatically digested cells that attached to the flask surface after 3 days (in α-MEM with 10% FBS) and reached 80% confluency were dissociated and encapsulated in a gel bead and loaded to GemCode Instrument (10X Genomics) which generated barcoded single-cell droplets. Sequencing was performed with standard protocol using 10X Single Cell 3’ v2 and 10X chromium system. The library was sequenced with Nova PE150. In total, 12 158 cells were sequenced in one run. R package Seurat (version 2.3.0)^18^ was leveraged for subsequent quality control and clustering analysis. Cells expressing <200 or >5000 genes were filtered out for exclusion of noncell or cell aggregates. Cells with a percentage of mitochondrial genes >0.05 were also filtered out. After quality control, 11 878 cells were included in subsequent analysis. After log-normalizing the data, principle component analysis was performed for dimension reduction. Clusters generated with the first 10 principal components were visualized with t-distributed stochastic nearest neighbor embedding. Expression of selected genes was plotted with Seurat function FeaturePlot.

Adult subcutaneous adipose tissue stromal vascular cells in published data sets (GSM3717978^19^ and E-MTAB-6677^20^) were analyzed with corresponding methods described in the literature. Clustering-specific markers for each mesenchymal stem cell population were found with MSC population subseted Seurat (version 3) object.

### Pseudotime Trajectory Analysis

Pseudotime trajectory was plotted with R package monocle version 2.4 with default settings. Pseudotime ordering was performed using function reduceDimension with max_components set at 2 and reduction_method set as DDRTree. Cells that express *Acta2* but do not express *Myl6*, *Cnn1*, or *Myh11* were set as starting point of pseudotime. Significant genes are obtained with function differentialGeneTest (fullModelFormulaStr =~Pseudotime) and plotted with function plot_pseudotime_heatmap (num_clusters =3). In the heatmap, predicted values generated by function genSmoothCurves were plotted along 100 evenly spaced pseudotime values.^21^ Genes included in Kyoto encyclopedia of genes and genomes term TGF-β signaling or transcription factors (list obtained from transcription factor database^22^) were intersected with the 3 significantly changed gene modules and presented as heatmap. Branch point analysis was performed with BEAM function.

### Smooth Muscle Differentiation

PV-ADSCs were seeded on gelatin-coated flasks and differentiated with medium (α-MEM with 10% FBS and 5 ng/mL TGF-β1 [R&D systems]) for indicated time. Leptin (Peprotech, 450-31) or IGFBP-2 (R&D Systems, 797-B2-025) at indicated concentrations were used to manipulate differentiation.

### RFP Labeling of Cells

Lentiviral particles used to label PV-ADSCs with RFP (red fluorescent protein) were generated with LV H2b_RFP plasmid^23^ (a gift from Elaine Fuchs, Addgene, 26001).

### Subcutaneous Matrigel Plug Assay

Subcutaneous Matrigel plug assay experiments were conducted as described.^6,24,25^ PV-ADSCs were differentiated for 5 days with αMEM with 10% FBS 5 ng/mL TGF-β1. Mouse MS1 ECs (ATCC, CRL-2279) were prepared. Differentiated PV-ADSCs and mouse ECs were mixed in a 1:1 ratio in 100 μL Matrigel and injected subcutaneously to mice. The plugs were harvested 14 days after the injection for immunostaining and H&E staining. To track the PV-ADSCs, RFP-labeled cells were used.

### Cell Transplantation

Mouse vein segments were isografted into carotid arteries of C57BL/6J mice.^26^ RFP-labeled PV-ADSCs in culture (10^6^ cells) were seeded onto the adventitial side to envelope the vein grafts. Vein graft transplantation without cell wrapping was used as control. Grafted tissue fragments were harvested 2 weeks postsurgery and stained with H&E and immunofluorescent markers.

### ^1^H Nuclear Magnetic Resonance Metabolomics Analysis

Undifferentiated ADSCs and ADSCs cultured in differentiation medium (α-MEM with 10% FBS and 5 ng/mL TGF-β1) for 1 day were harvested and frozen in liquid nitrogen. Eight samples were acquired in each treatment and 1H nuclear magnetic resonance metabolomics was performed using method published with modifications.^27^

### Gas Chromatography-Mass Spectrometry Metabolomics Analysis

Undifferentiated ADSCs, ADSCs differentiated for 4 days, cells treated with miRNA mimic negative control or miR-378a-3p mimics were harvested, frozen in liquid nitrogen before analysis. Extraction of metabolites was done using a published protocol with modification.^28^

### Metabolomics Data Processing

Annotated metabolites and correspondent abundance were normalized to the total level of metabolites. Data scaling was mean-centered and divided by SD of each variable. Orthogonal projection to latent structures analysis^29^ and heatmap of various metabolites were obtained from MetaboAnalyst software.^30^

### Transfection of MiRNA Mimics, MiRNA Inhibitors, and SiRNAs

PV-ADSCs with 70% confluence were transfected with miRNA mimics, inhibitors or siRNAs (Thermo Fisher) with Lipofectamine RNAiMAX (Thermo Fisher). After optimization, the concentrations of miRNA mimics, miRNA inhibitors, and siRNAs were respectively 12.5, 60, and 12.5 nmol/L.

### Oxygen Consumption Rate and Extracellular Acidification Rate Measurements

Oxygen consumption rate (OCR) and extracellular acidification rate are measured with the Seahorse XF-24 extracellular flux analyzer (Seahorse Bioscience). PV-ADSCs with indicated treatments and corresponding controls were plated on XF-24 microplate coated with gelatin one day before the assay. XF Cell Mito Stress Kit was used to study the mitochondrial metabolism. OCR and extracellular acidification rate at basal level and after metabolic perturbations with the addition of 1 μmol/L oligomycin, 1 μmol/L carbonyl cyanide-p-trifluoromethoxyphenylhydrazone, and 1 μmol/L rotenone and anti-mycin A were measured. Calculations were obtained with the Agilent Seahorse Wave Software for Agilent Seahorse XF analyzers (Seahorse Bioscience).

### Statistical Analysis

Data with 5 or more experiment repeats passed KS normality test that determines data normality and the *F* test that assesses homogeneity of variance. Unpaired and 2-tailed Student *t* test were applied to analyze data between 2 groups. Data were expressed as mean±SD using GraphPad Prism 6. Comparisons across multiple groups with 5 experiment repeats per group were assessed with 1-way ANOVA test, followed by Bonferroni post hoc analysis. Comparisons across multiple groups with 3 experiment repeats per group were assessed with the Kruskal-Wallis test, followed by Bonferroni post hoc analysis. Experiment repeats in each group were specified in the figure legends. *P*<0.05 was considered statistically different.

### Data Availability

Clustering result of primary PV-ADSC scRNA-seq data has been made public on the Automated Single-cell Analysis Pipeline website (https://asap.epfl.ch) with the project name PV-ADSC scRNA-seq. Raw sequencing data of primary PV-ADSCs are available in Gene Expression Omnibus (GSE132581). Additional data are available from the corresponding author on request.

## Results

### Characterization and Localization of Stem Cells in the PVAT

**Figure 1.**
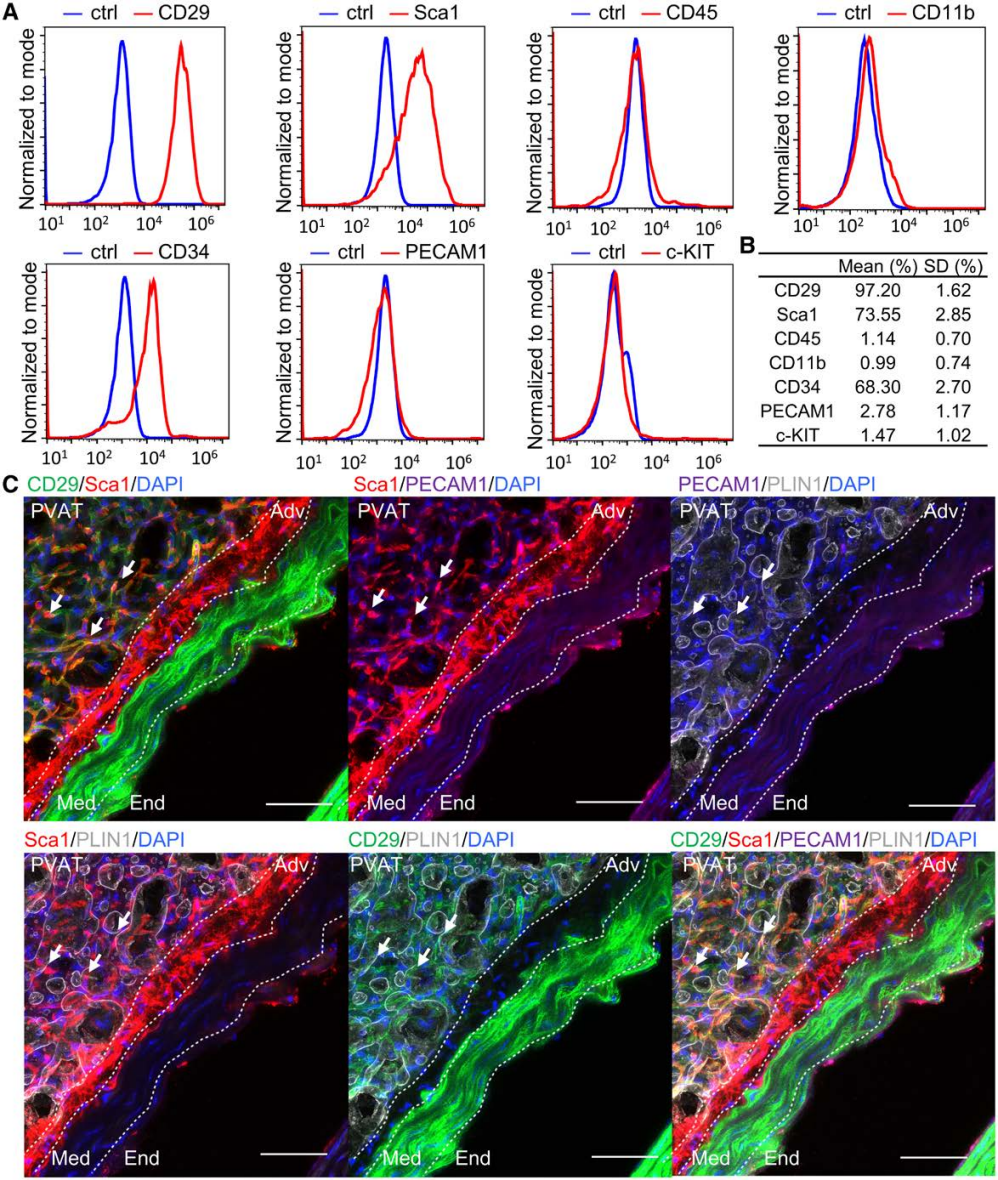
**Characterization of perivascular adipose tissue-derived mesenchymal stem cells (PV-ADSCs).** **A** and **B**, Representative histograms of flow cytometry analysis of cultured PV-ADSCs and the percentage of indicated phenotypic markers (n=3). Gating is set for the IgG control to be between 0.5% and 1%. **C**, Immunofluorescent staining of the adipose tissue surrounding the mouse aorta with CD29, Sca-1 (stem cell antigen 1), PECAM1 (platelet and endothelial cell adhesion molecule 1), PLIN-1 (perilipin-1), and 4′,6-diamidino-2-phenylindole (DAPI; n=3). Arrows indicate the CD29^+^/Sca1^+^/PECAM1^−^/PLIN1^−^ cells. The border between End (endothelium), Med (media), Adv (adventitia), and perivascular adipose tissue (PVAT) is drawn with dashed line. Scale bar, 50 μm. Ctrl indicates IgG isotype control.

MSCs exist in almost all organs of the body.^31^ ADSCs from the subcutaneous fat exhibit potential for tissue remodeling with multilineage differentiation capacities and immunomodulation effect.^32^ Thus, we hypothesized that MSCs also reside in the PVAT. To examine this, the adipose tissue surrounding mouse thoracic aorta was enzymatically digested and cultured in vitro. In the isolated adipose tissue, similar coverage of PLIN-1 (perilipin-1), an adipocyte marker with cell nucleus as well as minimal staining of PLIN-1 in remaining adventitia confirms minimal contamination of adventitial cells (Figure I in the online-only Data Supplement), although slight contamination could not be excluded given the proximity of periaorta adipose tissue and adventitia. More than 95% of the cells express MSC marker CD29 and 73.55±2.85% express mouse stem cell marker Sca1 (Figure 1A and 1B). CD34, which is also an MSC marker,^33^ displayed high expression in around 68.30±2.70% of the cultured cells (Figure 1A and 1B). Furthermore, only 1% to 3% of the cells express hematopoietic marker CD45, macrophage marker CD11b, progenitor marker c-Kit, and endothelial marker PECAM1 (platelet and endothelial cell adhesion molecule 1; Figure 1A and 1B). In addition, around 63% of the cells expressed pericyte or fibroblast marker PDGFRa and >95% expressed CD44 (Figure IIA in the online-only Data Supplement). Furthermore, the cells isolated from the PVAT exhibited adipogenic and osteogenic differentiation capacities (Figure IIB through IIE in the online-only Data Supplement). Thus, in consistency with the in vitro definition of MSCs proposed by the International Society for Cellular Therapy,^34^ phenotypic analysis, and multilineage differentiation capacities confirmed the existence of MSCs within the periaorta adipose tissue. These cells are referred to as PV-ADSCs below.

**Figure 2.**
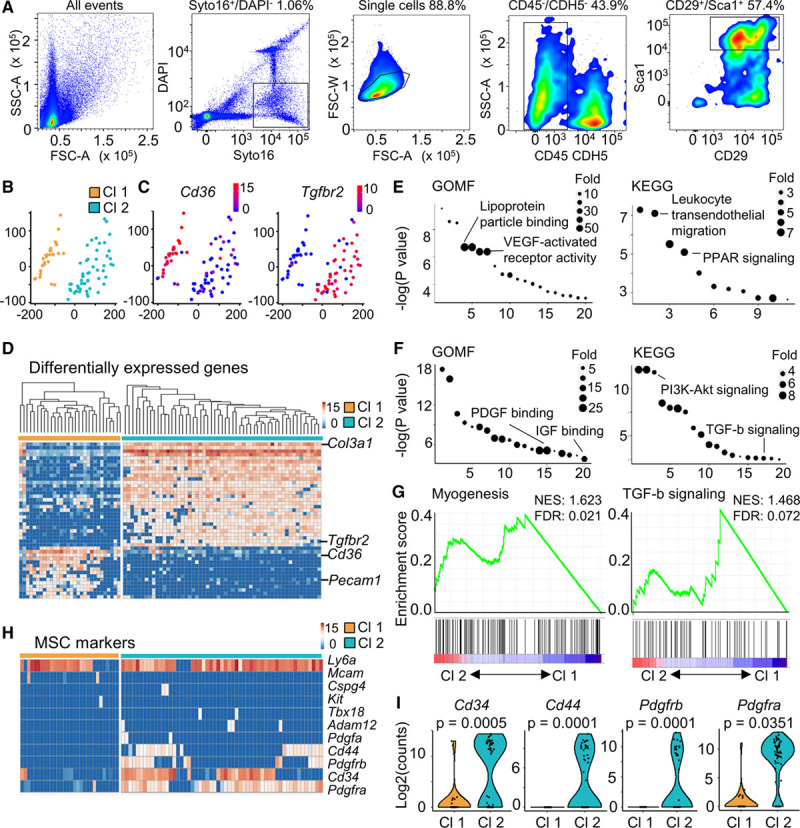
**Single-cell RNA-sequencing (scRNA-seq) reveals 2 distinct clusters in perivascular adipose tissue-derived mesenchymal stem cells (PV-ADSCs).** **A**, Sorting gate for scRNA-seq was set as live nucleated cells (Syto16^+^/DAPI^−^), single cells, CD45^−^/CDH5^−^ cells and CD29^+^/Sca1^+^ cells. **B**, Scatter plot obtained from multidimensional scaling (MDS) analysis showed 2 distinct clusters. Color scale, log2(gene counts). **C**, Expression of representative genes for cluster 1 (*Cd36*) and cluster 2 (*Tgfbr2*). **D**, Heatmap of marker genes in each cluster. Color scale, log2(gene counts). Full list of genes was in Table I in the online-only Data Supplement. **E** and **F**, Gene ontology (GO) and Kyoto encyclopedia of genes and genomes (KEGG) pathway analysis of the top 200 genes (by *P* value) upregulated in Cluster 1 (**E**) and Cluster 2 (**F**). Full list of GO terms and KEGG pathways was in Table II in the online-only Data Supplement. **G**, Enrichment of gene sets including Myogenesis (Molecular Signature) and TGF (transforming growth factor)-β signaling (KEGG). **H**, Expression heatmap of frequently used mesenchymal stem/stromal cell (MSC) markers. Color scale, log2(gene counts). **I**, Expression of frequently used MSC markers in the 2 clusters was shown as violin plot. Cl 1 indicates cluster 1; Cl 2, cluster 2; DAPI, 4′,6-diamidino-2-phenylindole; FDR, false discovery rate; GOMF, gene ontology molecular function; NES, normalized enrichment score; and PPAR-γ, peroxisome proliferator-activated receptor-γ.

After characterization of the phenotypic markers, the in situ localization of PV-ADSCs was studied. To exclude ECs and adipocytes from the PV-ADSC population, staining of endothelial marker PECAM1 and adipocytes marker PLIN-1 was performed. MSC marker CD29 was used because of its consistent and high expression in primary periaorta adipose tissue cells (Figure 2A) and cultured PV-ADSCs (Figure 1). As adventitial Sca-1^+^ cells play a crucial role in neointima formation and vascular pathogenesis,^35^ Sca-1 expression in CD29^+^/PECAM1^−^/PLIN1^−^ cells was examined. The results showed that PVAT contains a population of CD29^+^/PLIN1^−^/PECAM1^−^ cells and that some of the cells coexpress Sca-1 (Figure 1C). To conclude, a CD29^+^/Sca1^+^/PLIN1^−^/PECAM1^−^ PV-ADSC population was identified in the PVAT.

### ScRNA-Seq of PV-ADSCs Reveals 2 Distinct Clusters

Heterogeneity has been a long-standing question in the research field of MSCs.^34^ To achieve a high-resolution phenotyping of the PV-ADSCs, scRNA-seq was used. Among the enzymatically digested periaorta adipose tissue cells (freshly isolated cells without in vitro culture), single live cells were sorted for CD45^−^/CDH5^−^ population to exclude the hematopoietic and endothelial lineages (Figure 2A). Subsequently, scRNA-seq was performed in CD29^+^/Sca1^+^ cells (60.15±3.17% in nonimmune nonendothelial cells; Figure 2A; Figure IIIA in the online-only Data Supplement). Selection of CD29 and Sca1 as the positive markers of PV-ADSCs was supported by our previous phenotypic and in situ characterization and previous publications.^20,36^ Interestingly, Dpp4^+^ interstitial progenitors in mouse subcutaneous inguinal white adipose tissue showed an enrichment of Sca-1 compared with committed Icam1^+^ proadipocytes (Figure IIIB in the online-only Data Supplement), which further supported the use of Sca-1 for enrichment of ADSCs.^19^

Among the 94 single cells captured, 85 cells with the number of aligned reads >800 000 were selected for further analysis (Figure IIIC in the online-only Data Supplement). Two distinct clusters (cluster 1 and cluster 2) were identified which contained 30 and 55 cells, respectively (Figure 2B). Cluster 2 featured the expression of *Tgfbr2*, an important component of TGF-β signaling pathway (Figure 2C), whereas cluster 1 exclusively expressed *Pecam1* (Figure IIIH in the online-only Data Supplement), an endothelial marker and *Cd36* (Figure 2C and 2D), which is important for fatty acid uptake. The signature genes for clusters 1 and 2 were illustrated as a heatmap (Figure 2D). Important signaling pathways in cluster 1 included VEGF (vascular endothelial growth factor)-activated receptor activity and PPAR signaling (Figure 2E). The feature signaling pathways for cluster 2 were those essential in smooth muscle differentiation, such as PDGF binding, IGF (insulin-like growth factor) binding, PI3K (phosphatidylinositol 3-kinase)-Akt (AKT serine/threonine kinase 1) signaling, and TGF-β signaling (Figure 2F). Furthermore, gene sets of myogenesis and TGF-β signaling presented a positive correlation with cluster 2 (Figure 2G). The results implied that cluster 1 cells display angiogenic potential, whereas cluster 2 cells demonstrate enriched expression of genes involving pathways that are important in SMC differentiation.

Single-cell level examination of MSC or pericyte markers including *Mcam*,^31^ *Tbx18*^37^, *Cspg4*,^31^ and *Pdgfra*,^36^ etc, revealed substantial heterogeneity at a high resolution (Figure 2H). High expression of *Ly6a* was observed in both clusters, while other markers, such as *Cd34*, *Cd44*, *Pdgfra*, and *Pdgfrb*, were mainly expressed in cluster 2 (Figure 2I). Heterogeneous expression of stemness markers, such as *Klf4* and *Myc*,^38^ was also observed (Figure IIID in the online-only Data Supplement). Additionally, the cells in cluster 1 demonstrated exclusive expression of adipogenesis markers *Fabp4* and *Pparg* (Figure IIIE and IIIF in the online-only Data Supplement). Endothelial marker *Cdh5* was also mainly expressed in cluster 1 cells (Figure IIIG and IIIH in the online-only Data Supplement). No cell expressed SMC markers, including *Cnn1*, *Tagln*, *Acta2*, and *Myh11* (not shown). Interestingly, fibroblast markers such as *Vimentin* demonstrated heterogeneous expression in primary PV-ADSCs (Figure IV in the online-only Data Supplement). Relatively limited heterogeneity resulted from the relatively small number of cells analyzed was complimented by analysis of published data sets which offered clue of subcutaneous stromal vascular cell hierarchy (Figure V in the online-only Data Supplement).^19,20^ To conclude, scRNA-seq allowed unprecedented direct visualization of MSC heterogeneity, revealing 2 distinct clusters with discrete signature gene sets and signaling pathways. Multiple gene sets involved in SMC differentiation were enriched in cluster 2.

### Pseudotime Analysis of Cultured PV-ADSCs Uncovers an SMC Differentiation Trajectory

While scRNA-seq of primary PV-ADSCs reflected their function and heterogeneity in vivo, further scRNA-seq of cultured PV-ADSCs offered an opportunity to examine a larger number of cells. In total, 12 158 cells were detected, with a median 2602 genes per cell. After quality control by including cells with gene number between 200 and 5000 and filtering out cells with fraction of mitochondrial genes higher than 0.05, 11 878 cells were leveraged for subsequent analysis (Figure VIA and VIB in the online-only Data Supplement). Consistent with our previous flow cytometry data (Figure 1A), cultured PV-ADSCs exhibited minimal level of *Cdh5* expression with only 0.48% of the cells positive for *Cdh5* (Figure VIC and VIE in the online-only Data Supplement). On the contrary, *Tgfbr2* and *Col3a1*, both marker genes of cluster 2 in primary PV-ADSCs, displayed high expression in cultured PV-ADSCs with 29.26% and 93.79% of the cells positive for these markers, respectively (Figure VIC and VIE in the online-only Data Supplement). Interestingly, 6.23% of the cells expressed mature SMC marker *Myh11* (Figure VID and VIE in the online-only Data Supplement). Additionally, most of the 15 cluster 1 marker genes displayed expression in <5% cultured cells, whereas most of the cluster 2 marker genes were expressed in >25% of the cultured cells (Figure VIF in the online-only Data Supplement).

**Figure 3.**
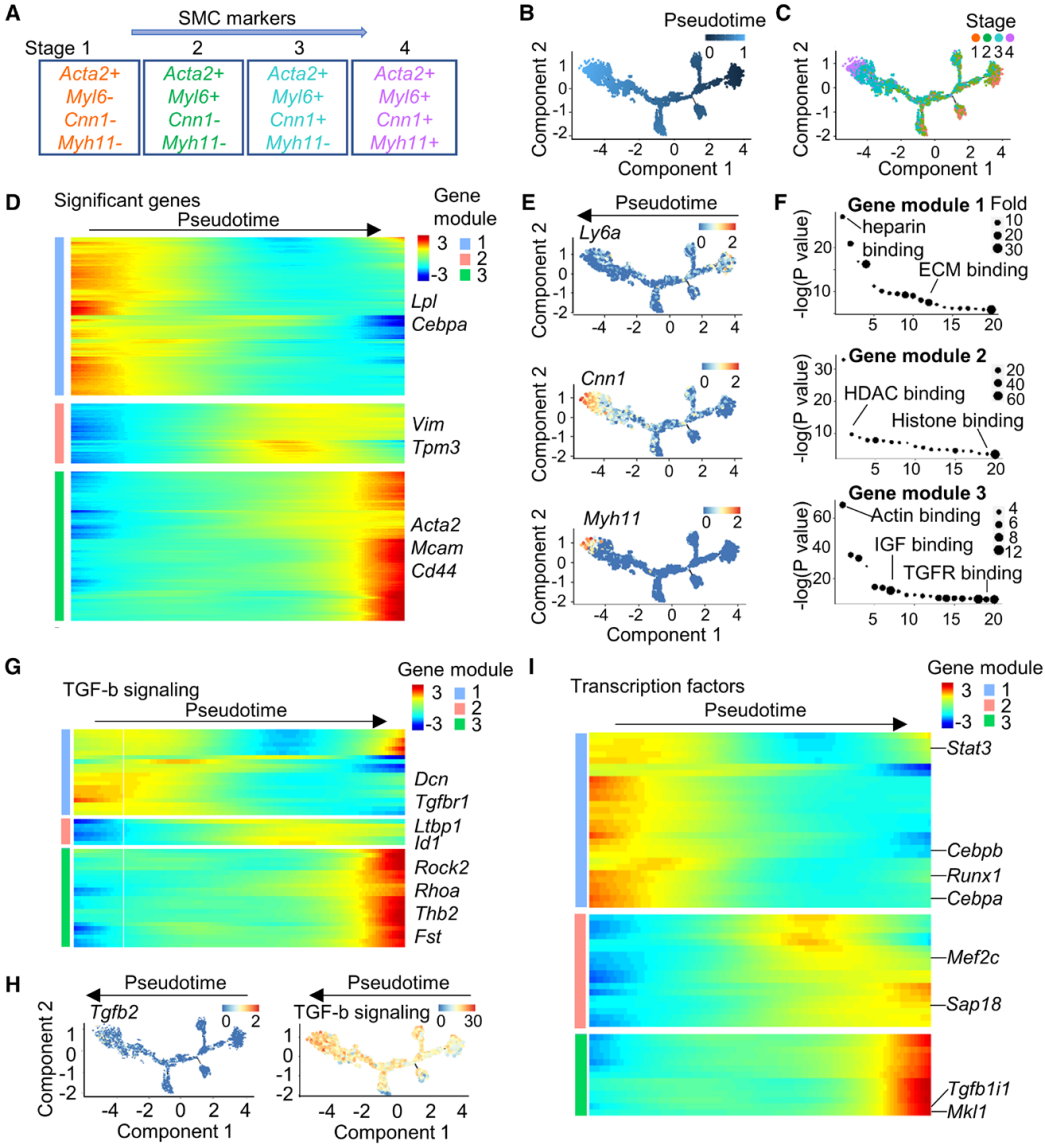
**Pseudotime trajectory of cultured perivascular adipose tissue-derived mesenchymal stem cells (PV-ADSCs).** **A**, Cell stages expressing early to late smooth muscle cell (SMC) markers. **B**, Pseudotime trajectory of cultured PV-ADSCs with DDRTree method for dimension reduction. Color scale, pseudotime. **C**, Cell ordering from different differentiation stages along the pseudotime trajectory. **D**, Heatmap of the top 1000 (by *Q* value) significantly changed genes in 3 gene modules. **E**, Expression of *Ly6a*, *Cnn1*, and *Myh11* along the trajectory. Color scale, log gene expression. **F**, Gene ontology (molecular function) analysis of each gene module from (**D**). Full list was in Table III in the online-only Data Supplement. **G**, Expression of significantly changed genes from TGF (transforming growth factor)-β signaling pathway (KEGG) along pseudotime. Full list was in Table I in the online-only Data Supplement. Color scale, log gene expression. **H**, *Tgfb2* expression and total level of significantly changed genes from TGF-β signaling along the pseudotime trajectory. Color scale, log gene expression. **I**, Expression of significantly changed transcription factors along the pseudotime trajectory. Full list was in Table I in the online-only Data Supplement. Color scale, log gene expression.

Pseudotime trajectory analysis was used to inspect progression of continuous cell states. It is noted here that the trajectory here was not complimented by real differentiation time points and thus offers only suggestions of differentiation mechanism, which requires further experimental proof. Because the PV-ADSCs were kept in basal conditions without additional stimulation factor towards adipocytes or osteocytes, minimal expression of markers for these cell types were expressed (Figure VIIA in the online-only Data Supplement). Cultured PV-ADSCs displayed different level of *Acta2, Myl6, Cnn1*, and *Myh11* expression (Figure 3A; Figure VIIB in the online-only Data Supplement). Pseudotime analysis ordered cells expressing different levels of SMC markers in a trajectory (Figure 3B). Mature SMCs (stage 4) were located towards the termini of the trajectory, which is partly a validation for the constructed trajectory (Figure 3C). Significantly changed genes along the pseudotime trajectory were assigned to 3 gene modules (Figure 3D). In the downregulated gene module were *Lpl* (Figure 3D), an enzyme important for lipid metabolism,^39^ stem cell marker *Ly6a* (Figure 3E), and fibroblast marker *Dcn* (Figure VIIB in the online-only Data Supplement). *Vim* and *Tpm3* were from gene module 2 and upregulated along the pseudotime, peaking in the middle of the trajectory (Figure 3D). SMC marker *Acta2*, pericyte marker *Mcam*, and MSC marker *Cd44* were in gene module 3, reaching highest expression towards the end of trajectory (Figure 3D). For the upregulated genes (gene module 2 and 3), essential pathways for SMC differentiation such as HDAC (histone deacetylase) binding, histone binding, acting binding, IGF binding, and TGFR binding were enriched (Figure 3F). In the TGF-β signaling pathway, the upregulation of *Tgfb1* (in gene module 2), *Rock2*, and *Rhoa* (in gene module 3) along pseudotime trajectory further underlines the importance of this pathway in SMC differentiation (Figure 3G). In particular, *Tgfb2* and also the total level of significantly changed genes in TGF-β signaling were increased during differentiation (Figure 3H). Importantly, along the trajectory, *Cebpb*, which is crucial for adipogenesis, and *Runx1*, which drives osteocyte differentiation,^40^ decreased (Figure 3I). It is noted that pseudotime heatmap plots predicted values generated by function genSmoothCurve in monocle (version 2) along 100 evenly spaced pseudotime values rather than real expression values.^21^ Cells located at the branches of the trajectory including states 8, 7, 5, and 9 demonstrated enriched expression of genes involved in gene ontology terms antioxidant activity, laminin binding, hormone receptor binding, and IL (interleukin)-1 activity receptor binding respectively, which is consistent with the broad spectrum of function reported for mesenchymal stem cells such as immunomodulation (Figure VIII in the online-only Data Supplement).^41^

### PV-ADSCs Participate in Vascular Remodeling In Vivo and Differentiate Towards SMCs In Vitro

Both adventitial progenitors and PV-ADSCs are located close to the aorta, among which adventitial progenitors have been demonstrated to participate in vascular remodeling.^7^ To better characterize PV-ADSCs, comparison of their single-cell level transcriptomic signature with adventitial mesenchyme cells (Adventitial Mesen I-IV clusters)^42^ were performed. Cluster-defining genes for Adv-Mesen cells were enriched in cluster 2 cells of primary PV-ADSCs, with Mesen II cluster markers most highly expressed (Figure IXA in the online-only Data Supplement). Additionally, only cluster 2 markers were highly expressed in Adv-Mesen cells and their slight enrichment in Mesen II cluster was also observed (Figure IXB in the online-only Data Supplement). Comparison of Sca1^+^ (normalized expression >1) cells from Adv-Mesen cells and cultured PV-ADSCs was further conducted. Interestingly, *Ccl2* (C-C motif chemokine ligand 2), which proved important for the proinflammatory function of adventitial Sca1^+^ cells,^42^ was among the genes with high variation in both groups (Figure IXC from online-only Data Supplement). Various genes were differentially expressed in Sca1^+^ Adv-Mesen cells and cultured PV-ADSCs, among which fibroblast marker *Dcn* (decorin) and actin filament regulating *Gsn* (gresolin) were enriched in Adv-Mesen cells, and *Timp1* (tissue inhibitor of metalloproteinases 1) and *S100a4* (S100 calcium binding protein A4) were highly expressed in cultured PV-ADSCs (Figure IXD and IXE in the online-only Data Supplement). In terms of pathway analysis, genes expressed higher in cultured PV-ADSCs displayed enrichment of gene ontology terms actin binding and actin filament binding, whereas those selectively expressed in Adv-Mesen cells were enriched for gene ontology term chemokine activity and antioxidant activity (Figure IXF in the online-only Data Supplement). However, it should be noted that cultured PV-ADSCs and primary Adv-Mesen cells were compared. Cell culture conditions might introduce transcriptomic difference. Collectively, cluster 2 PV-ADSCs displayed certain similarity with adventitial Sca1^+^ cells, whereas transcriptomic difference was more prominent.

**Figure 4.**
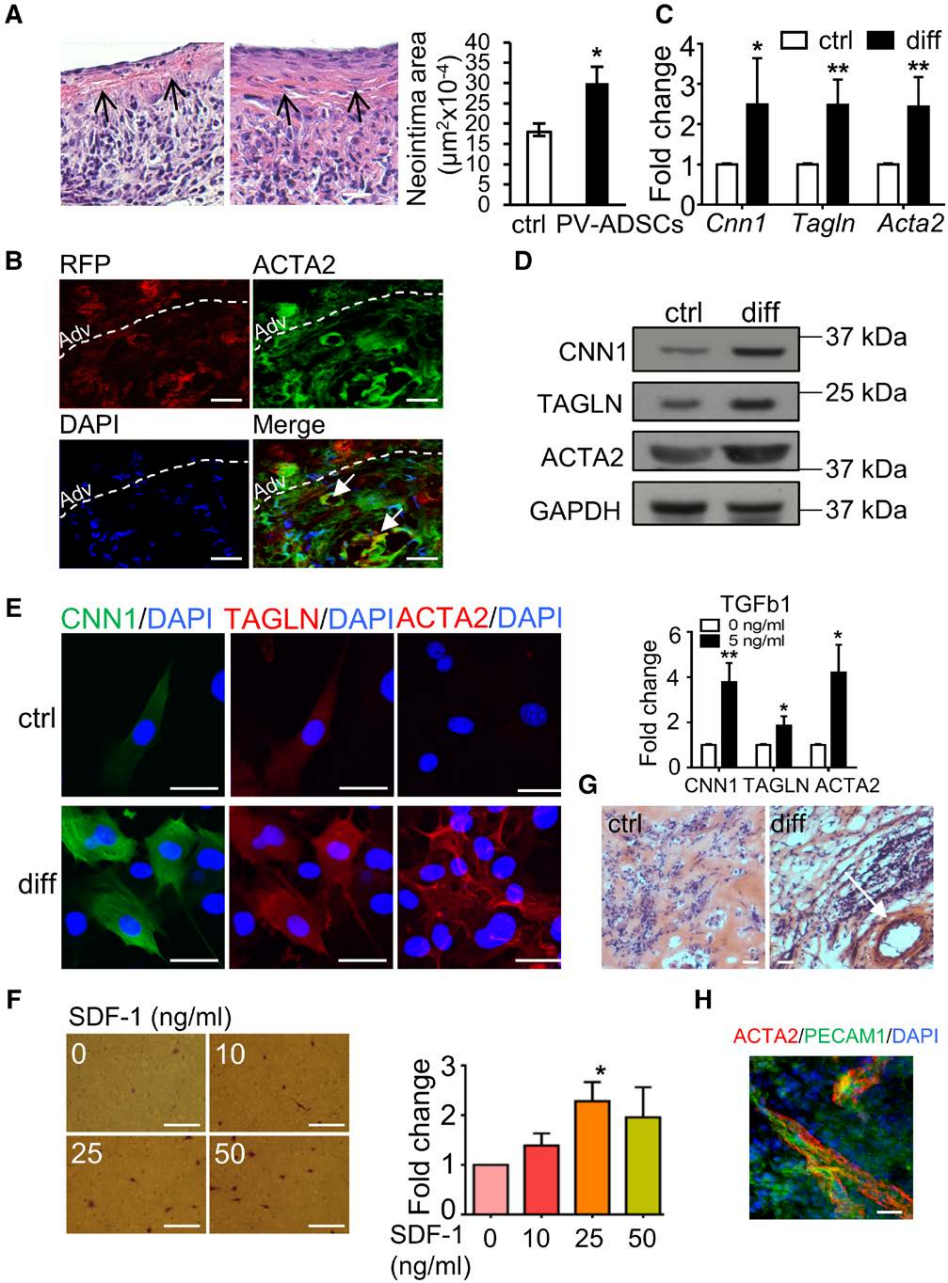
**Perivascular adipose tissue-derived mesenchymal stem cells (PV-ADSCs) differentiate towards smooth muscle cells (SMCs) in vivo and in vitro.** **A**, H&E staining of the vein graft harvested 4 wk after the transplantation. Neointimal area was calculated against injury-only control (n=4). Arrow indicates the boundary between neointima and media layer. **B**, Vein grafts harvested 1 wk after the transplantation were stained with RFP (red fluorescent protein; red) and SMC marker ACTA2 (smooth muscle α actin; green) (n=4). Arrows indicate cells positive for both markers. Dashed line indicates boundary of adventitia and neointima. Scale bar, 20 μm. **C**–**E**, In vitro cultured PV-ADSCs were differentiated towards SMCs in differentiation medium for 5 d (diff) and then harvested for quantitative polymerase chain reaction (qPCR; **C**; n=4), Western blot (**D**; n=3) and immunofluorescent staining (**E**; n=3) for SMC markers. Cells cultured without TGF (transforming growth factor)-β1 (ctrl) served as control. Scale bar, 20 μm. **F**, Chemotaxis of PV-ADSCs in response to an increasing gradient of SDF-1 (stromal cell-derived factor 1) in an 8.0-μm transwell system was identified by applying 1% crystal violet staining after 16-h incubation (n=3). Scale bar, 50 μm. **G**, H&E staining of subcutaneous *Matrigel* plug containing PV-ADSCs-derived SMCs and endothelial cells (ECs; diff) in comparison with control group (ctrl) (n=4). **H**, Immunostaining of the tube-like structure in *Matrigel* plug with ACTA2 and PECAM1 (platelet and endothelial cell adhesion molecule 1; n=4). Scale bar, 50 μm. Data are presented as mean±SD. *P<0.05, **P<0.01. Adv indicates adventitia. CNN1 indicates calponin; DAPI, 4′,6-diamidino-2-phenylindole; and TAGLN, smooth muscle protein 22-α.

To explore whether PV-ADSCs participate in vascular remodeling in vivo, a vein graft model was used. Transplantation of cultured PV-ADSCs to the adventitial side of the vein graft significantly promoted the neointima formation (Figure 4A). RFP signal in the neointimal area indicated the migration of the RFP-labeled PV-ADSCs from the adventitial side to the neointima (Figure 4B). Numerous RFP-positive cells expressed SMC marker ACTA2 (smooth muscle α actin), implying that the contribution of PV-ADSCs to neointima formation might be partly through SMC differentiation (Figure 4B). Fibroblast marker Vimentin was also expressed in some RFP-positive cells (Figure XA in the online-only Data Supplement). Notably, no colocalization of RFP with macrophage marker CD68 was observed, suggesting that participation of PV-ADSCs in vascular remodeling was not through macrophage differentiation (Figure XB in the online-only Data Supplement).

After establishing that PV-ADSCs contribute to vascular remodeling partly through SMC differentiation in vivo, their potential to differentiate towards SMCs in vitro was next explored for subsequent mechanism study. TGF-β1 was used as stimulus for differentiation, given the role of TGF-β1 signaling in SMC differentiation implied by scRNA-seq. On treatment with TGF-β1, PV-ADSCs differentiated towards SMCs, as displayed by the upregulation of SMC markers including Cnn1, Tagln, and Acta2 at the mRNA and the protein level (Figure 4C through 4E). Collagen gel contraction assay demonstrated better contractility of SMCs differentiated from PV-ADSCs (Figure XI in the online-only Data Supplement). Since brown adipose tissue has been gradually recognized as a secretory organ, we also checked the effect of IGFBP-2 (insulin like growth factor binding protein 2), an adipokine secreted predominantly by brown adipose tissue,^43^ on PV-ADSC differentiation towards SMCs. High concentration (250 ng/mL) IGFBP-2 promoted SMC differentiation with the presence of TGF-β1, whereas no effect was observed for leptin, an adipokine secreted mainly by white adipose tissue (Figure XIIA and XIIB in the online-only Data Supplement). Interestingly, PV-ADSCs also expressed high level of IGFBP-2 in comparison with ADSCs isolated from subcutaneous adipose tissue (Figure XIIC in the online-only Data Supplement). Additionally, PV-ADSC migration towards chemoattractant SDF-1 (stromal cell-derived factor 1) was also observed (Figure 4F), consistent with the notion that participation of PV-ADSCs in vivo involves complex processes, including differentiation and migration.^7^

To further investigate the function of SMCs differentiated from PV-ADSCs, the cells were mixed with mouse MILE SVEN 1 ECs in *Matrigel* and then injected subcutaneously in wild-type mouse to check their ability to participate in vasculogenesis with the assistance of mature ECs. As exhibited by H&E staining of the subcutaneous *Matrigel* plug, a relatively large tube-like structure with multiple layers was formed in the plug with differentiated PV-ADSCs and ECs, whereas it was not found in control group (Figure 4G). Immunostaining of the *Matrigel* plug showed proximal localization of ACTA2 and PECAM1, demonstrating that the tube-like structure was a vessel (Figure 4H). Altogether, functional SMCs can be derived from PV-ADSCs.

### Metabolic Reprogramming of PV-ADSCs During SMC Differentiation Induced by TGF-β1

**Figure 5.**
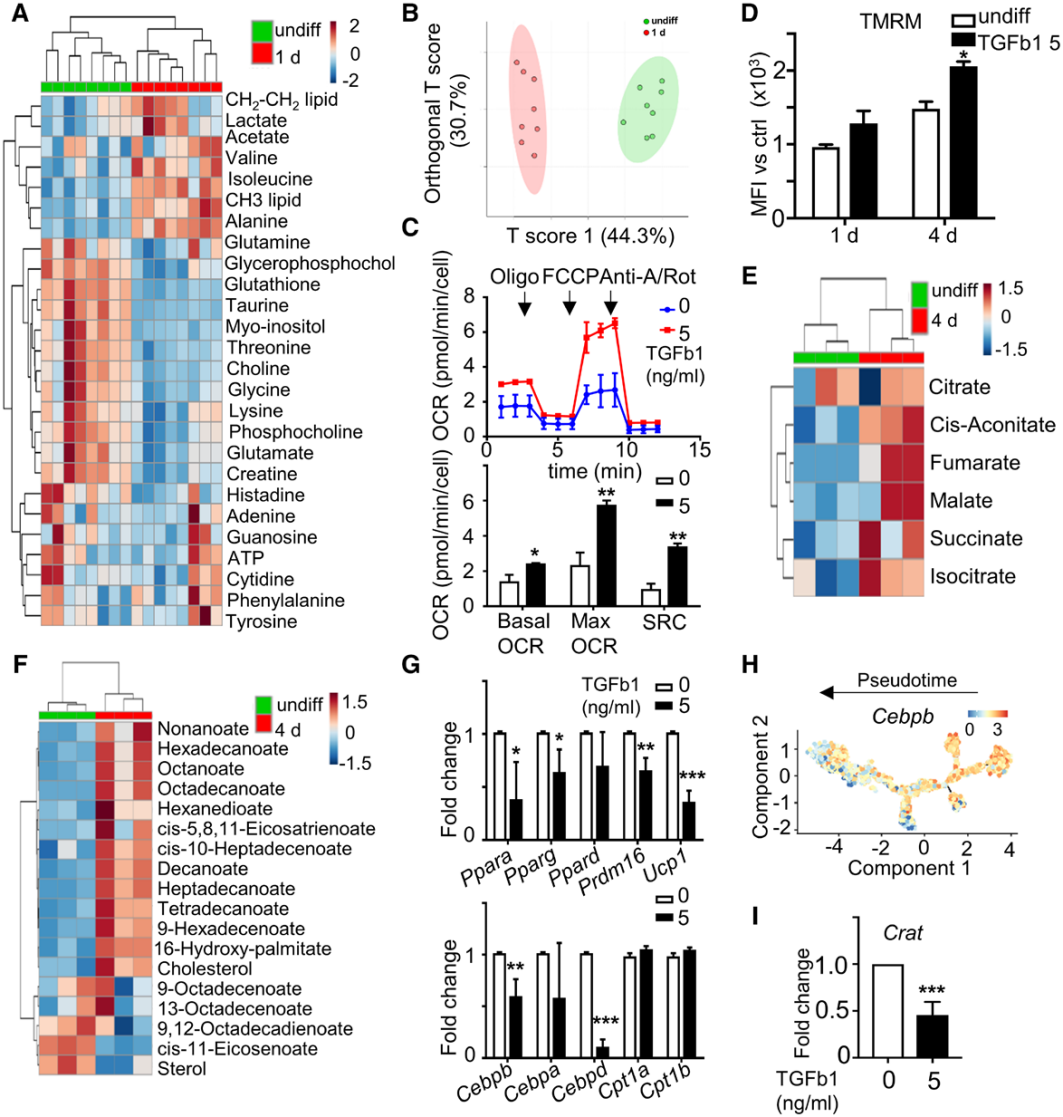
**Metabolic reprogramming of perivascular adipose tissue-derived mesenchymal stem cells (PV-ADSCs) during smooth muscle cells (SMC) differentiation.** **A** and **B**, Cellular metabolite abundance was detected with nuclear magnetic resonance (NMR) system in undifferentiated PV-ADSCs and PV-ADSCs differentiated for 1 d (n=8). **A**, Heatmap for metabolite levels. **B**, Orthogonal projection to latent structures. **C**, The oxygen consumption rate (OCR) measured in PV-ADSCs cultured with or without TGF (transforming growth factor)-β1 for 1 d (n=3). **D**, Live PV-ADSCs cultured in differentiation medium or basal growth medium for 1 and 4 d were stained tetramethylrhodamine, methyl ester, perchlorate (TMRM) to detect mitochondrial potential (n=3). **E** and **F**, Cellular metabolite abundance in undifferentiated PV-ADSCs or PV-ADSCs differentiated for 4 days was determined with gas chromatography-mass spectrometry (GC-MS) system (n=3). Heatmaps of metabolites involved in tricarboxylic acid (TCA) cycle (**E**) or lipid metabolism (**F**) were shown. **G**, Gene expression of genes important in lipid metabolism in PV-ADSCs treated with or without TGF-β1 for 2 d (n=4). **H**, Expression of *Cebpb* along the pseudotime trajectory. Color scale, log expression of gene. **I**, *Crat* mRNA level in PV-ADSCs treated with or without TGF-β1 for 2 d (n=4). Data are presented as mean±SD. *P<0.05, **P<0.01, and ***P<0.001. Anti-A/Rot indicates anti-mycin A/Rotenone; FCCP, carbonyl cyanide-4-phenylhydrazone; MFI, mean fluorescence intensity; and SRC, spare respiratory consumption.

Metabolic reprogramming in stem cells not only helps to meet the energy requirement but also drives various processes such as differentiation.^44^ To investigate metabolic changes in the SMC differentiation of PV-ADSCs induced by TGF-β1, nuclear magnetic resonance metabolomics was firstly performed. Abundance of various detected metabolites demonstrated notable changes during differentiation (Figure 5A). Orthogonal projection to latent structures analysis depicted the distinct metabolic status of differentiated PV-ADSCs (Figure 5B). Functionally, cells treated with TGF-β1 exhibited higher basal mitochondrial OCR, higher maximal mitochondrial OCR as well as stronger spare respiration capacity (Figure 5C). Consistently, higher mitochondrial potential was detected in differentiated PV-ADSCs (Figure 5D). These results implied that the mitochondrial oxidative metabolism was enhanced during differentiation.

Further characterization of the metabolic change was examined with gas chromatography-mass spectrometry metabolomics, which is more sensitive and detects more metabolites. Enrichment of metabolites from tricarboxylic acid cycle (Figure 5E) during differentiation indicated the increased mitochondrial activity, which was consistent with the upregulated OCR and mitochondrial potential. Fueled by the observation that mitochondrial activity was increased during differentiation, we postulated that the reactive oxygen species might influence SMC differentiation. However, SMC markers were not altered by the treatment of H_2_O_2_ (Figure XIIIA in the online-only Data Supplement) or the transfection of *Nox4* siRNA (Figure XIIIB in the online-only Data Supplement) which changed the reactive oxygen species level. Treatment of carbonyl cyanide-p-trifluoromethoxyphenylhydrazone which potently uncoupled the electron transduction and oxidative phosphorylation also displayed only mild effect on SMC marker expression (Figure XIIIC in the online-only Data Supplement). Notably, as indicated by the increased phosphoenolpyruvate and lactate level (Figure XIIID in the online-only Data Supplement), glycolysis was also upregulated during differentiation. Functional characterization of glycolysis showed the upregulation of both the basal glycolysis and glycolysis capacity in cells treated with TGF-β1 (Figure XIIIE in the online-only Data Supplement). However, glucose uptake did not show much change and glucose treatment did not have effect on the SMC marker expression (Figure XIIIF and XIIIG in the online-only Data Supplement).

More extensive examination of the gas chromatography-mass spectrometry metabolomics data revealed the upregulation of various lipids in differentiated PV-ADSCs (Figure 5F). The increased lipid together with the more active mitochondria suggested a potential change of lipid metabolism. Significant changes in *Ppara, Pparg, Prdm16, Ucp1, Cebpb*, and *Cebpd* were observed during SMC differentiation, supporting the hypothesis that lipid metabolism drastically reprogrammed during differentiation (Figure 5G). Changes of these markers along the trajectory and in real time further supported the reprogramming of lipid metabolism (Figure 5H; Figure XIV in the online-only Data Supplement). The change of various lipid levels led us to investigate the lipid oxidation process, which relies on the transmitochondrial transport of fatty acids regulated by carnitine acyltransferases including carnitine palmitoyltransferase 1 (*Cpt1*) and carnitine acetyltransferase (*Crat*).^45^ *Cpt1* is responsible for the transport of long-chain fatty acids, and *Crat* is responsible for the transport of short-chain fatty acids.^45^ *Cpt1a* and *Cpt1b* were not altered with the treatment of TGF-β1 for 2 days (Figure 5G), although *Cpt1a* displayed downregulation along trajectory (Figure XIV in the online-only Data Supplement). Moreover, *Crat* level was significantly downregulated first and then upregulated during SMC differentiation, which is supported by the early downregulation and late upregulation of *Crat* level along SMC trajectory (Figure 5I; Figure XIV in the online-only Data Supplement). Thus, significant metabolic reprogramming was induced during SMC differentiation and altered lipid metabolism implied the involvement of *Crat* in SMC differentiation.

### TGF-β1 Promotes SMC Differentiation via Inhibition of Crat

**Figure 6.**
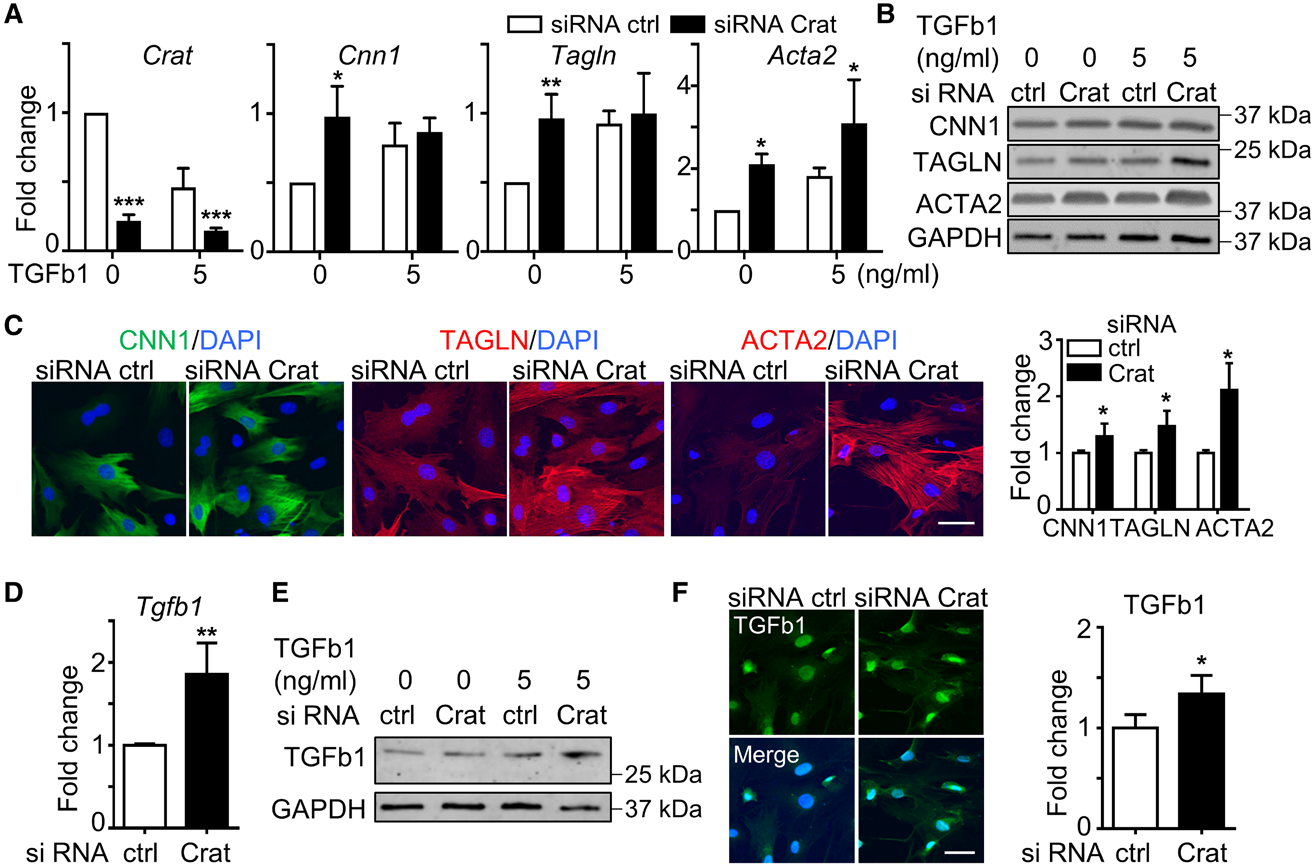
**Crat knockdown promotes smooth muscle cells (SMC) differentiation via upregulation of TGF (transforming growth factor)-β1.** **A–C**, Perivascular adipose tissue-derived mesenchymal stem cells (PV-ADSCs) treated with Crat siRNA with or without TGF-β1 for 2 d. **A**, *Crat* level was significantly downregulated after siRNA Crat transfection. Quantitative polymerase chain reaction (qPCR) also showed the induction of SMC markers (*Cnn1, Tagln*, and *Acta2*) with Crat knockdown. **B**, Protein level induction of SMC markers by Crat siRNA was demonstrated by Western blot. **C**, Immunostaining and statistical analysis showed the induction of SMC markers by Crat siRNA in medium without TGF-β1. **D–F**, TGF-β1 level after Crat siRNA transfection for 2 d was determined with qPCR (**D**), Western blot (**E**), and immunofluorescent staining (**F**). Results are cumulative (**A** and **D**) or representative (**B**, **C**, **E**, and **F**) of 3 independent experiments. Scale bar, 30 μm. Data are presented as mean±SD. *P<0.05, **P<0.01, and ***P<0.001. ACTA2 indicates smooth muscle α actin; CNN1, calponin; DAPI, 4′,6-diamidino-2-phenylindole; and TAGLN, smooth muscle protein 22-α.

In SMC differentiation, TGF-β1 altered the lipid profile and inhibited the level of Crat. To confirm the role of *Crat* in SMC differentiation, siRNA *Crat* was used. Quantitative polymerase chain reaction showed successful knockdown of *Crat* 2 days after *Crat* siRNA transfection (Figure 6A). SMC markers including *Cnn1, Tagln*, and *Acta2* were induced at the mRNA level (Figure 6A) by *Crat* siRNA transfection. Promotion of SMC differentiation by *Crat* knockdown was demonstrated at the protein level by Western blot (Figure 6B) and immunofluorescent staining (Figure 6C) 2 days after *Crat* siRNA transfection. The upregulation of TGF-β1 at the mRNA level (Figure 6D) and the protein level (Figure 6E and 6F) 2 days after *Crat* siRNA transfection identified the induction of TGF-β1 as the downstream mechanism of Crat-mediated SMC differentiation. To conclude, an interesting feedback control was identified between Crat inhibition and TGF-β1 induction during differentiation.

### MiR-378a-3p Induces Metabolic Reprogramming and Promotes SMC Differentiation

**Figure 7.**
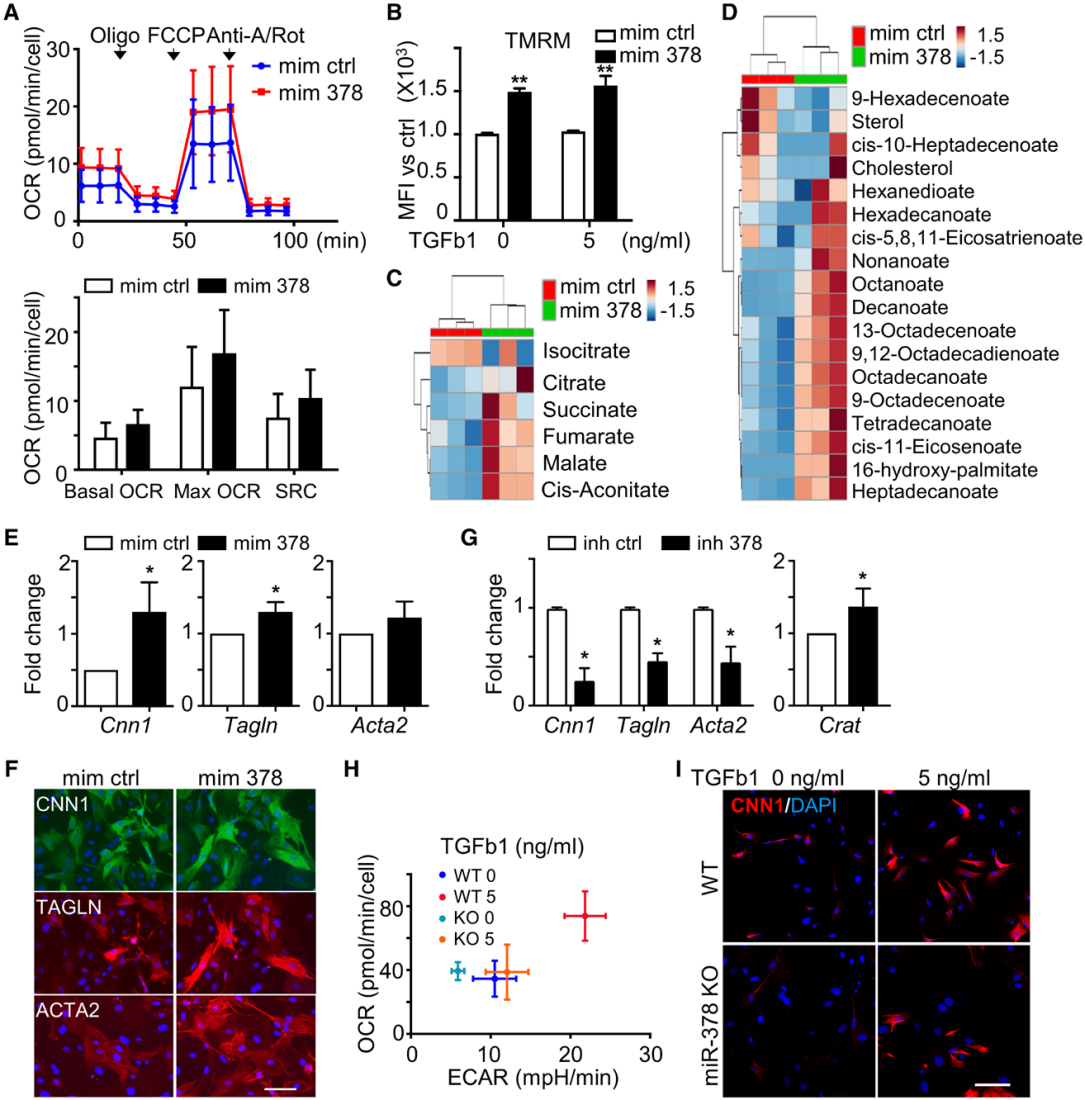
**MicroRNA (miR)-378a-3p induces metabolic reprogramming and promotes smooth muscle cells (SMC) differentiation.** **A**, The oxygen consumption rate (OCR) of perivascular adipose tissue-derived mesenchymal stem cells (PV-ADSCs) cultured in miR-378a-3p mimics or control for 2 d (n=3). **B**, Live PV-ADSCs treated with miR-378a-3p mimic for 2 d with or without TGF (transforming growth factor)-β1 were stained tetramethylrhodamine, methyl ester, perchlorate (TMRM) and analyzed for mean fluorescence intensity (MFI) with flow cytometry (n=3). **C** and **D**, Cellular metabolite abundance in PV-ADSCs treated with miR-378a-3p mimics for 1 d determined with gas chromatography-mass spectrometry (GC-MS) system (n=3). Heatmap of metabolites in tricarboxylic acid (TCA) cycle (**C**) or lipid metabolism (**D**). **E**, SMC marker mRNAs were induced by miR-378a-3p mimics after 2 d (n=3). **F**, Representative immunofluorescent staining (n=3) showed the upregulation of SMC markers by miR-378a-3p mimic treatment. **G**, Transfection of PV-ADSCs with miR-378a-3p inhibitor inhibited the level of SMC markers as shown by quantitative polymerase chain reaction (qPCR). The level of *Crat* was derepressed by miR-378a-3p inhibitor transfection (n=3). **H**, OCR and extracellular acidification rate (ECAR) were detected with Seahorse Mito stress tests in wild-type (WT) and miR-378a knockout (KO) ADSCs (n=3). **I**, WT and miR-378a KO ADSCs were treated with or without TGF-β1 for 2 d. Immunofluorescent staining showed the protein level of CNN1 (calponin; n=1). Scale bar, 100 µm. Data are mean±SD. *P<0.05 and **P<0.01. Anti-A/Rot indicates anti-mycin A/Rotenone; Crat, carnitine acetyltransferase; FCCP, carbonyl cyanide-4-phenylhydrazone; inh 378, miR-378a-3p inhibitor; inh ctrl, inhibitor control; mim 378, miR-378a-3p mimics; mim ctrl, mimics control; and SRC, spare respiratory capacity.

After establishing the change of the metabolic profile during differentiation process and the mechanism involved in SMC differentiation, potential therapeutic targets that could similarly alter the metabolic profile and drive SMC differentiation were investigated. Literature mining revealed that miR-378a-3p could target *Crat* and thus regulate fatty acid metabolism which was regulated by TGF-β1 in our SMC differentiation system.^46^ To study the role of miR-378a-3p in metabolic reprogramming and SMC differentiation, PV-ADSCs were transfected with miR-378a-3p mimics. In Seahorse Mito Stress tests, both the basal OCR and maximal OCR showed an increasing trend with miR-378a-3p mimic treatment compared with control (Figure 7A). Furthermore, the mitochondrial potential also increased (Figure 7B). Metabolite abundance detected by gas chromatography-mass spectrometry system demonstrated the enrichment of metabolites involved in the tricarboxylic acid cycle (Figure 7C) and upregulated level of various lipids, such as cholesterol (Figure 7D). Overall, the metabolic reprogramming induced by the miR-378a-3p mimics was similar to that induced by TGF-β1. Furthermore, miR-378a-3p mimics induced SMC differentiation with the significant upregulation of *Cnn1* and *Tagln* (Figure 7E). The induction of SMC gene expression was confirmed at the protein level (Figure 7F; Figure XVA in the online-only Data Supplement). On the contrary, miR-378a-3p inhibitor downregulated mRNA level of SMC markers (Figure 7G). In addition, Crat expression was also derepressed in PV-ADSCs after the transfection of miR-378a-3p inhibitors (Figure 7G).

Finally, TGF-β1 treatment could not induce an upregulation of OCR and extracellular acidification rate in ADSCs derived from miR-378a-3p knockout mice (Figure 7H), further confirming the importance of miR-378a in inducing metabolic reprogramming. Also, SMC differentiation capacity was significantly attenuated in ADSCs derived from miR-378a knockout mice as shown by the lower level of CNN1 (calponin) compared with ADSCs from wild-type mice after the treatment with TGF-β1 (Figure 7I; Figure XVB in the online-only Data Supplement). Interestingly, level of miR-378a-3p was downregulated by TGF-β1 treatment (Figure XVC in the online-only Data Supplement). In summary, metabolic reprogramming and SMC differentiation were induced by miR-378a-3p, which was an indispensable component for TGF-β1 to exert the metabolic regulation.

## Discussion

In this study, we characterized the transcriptomic profile of PV-ADSCs at a single-cell level and determined their role in vascular remodeling through their differentiation towards smooth muscle lineage which was accompanied by metabolic reprogramming. Substantial heterogeneity was observed at the single-cell level in primary PV-ADSCs. Moreover, through differentiation towards smooth muscle lineage, cultured PV-ADSCs were suggested to contribute to vascular remodeling in vein graft models. Mechanistically, SMC differentiation from PV-ADSCs was accompanied by a metabolic reprogramming which could be induced by miR-378a-3p.

To characterize the PV-ADSCs, scRNA-seq was performed and revealed 2 distinct clusters within the CD45^−^/CDH5^−^/CD29^+^/Sca1^+^ population, as well as the signature gene sets and feature signaling pathways for each cluster. However, attention needs to be paid to the markers used to identify the MSCs. About 60.15±3.77% of the CD45^−^/CDH5^−^ population were CD29^+^/Sca1^+^. Thus, the scRNA-seq result only covers this population and remaining cells necessitate further characterization. Furthermore, multiple cluster 1 markers including *Pecam1* and *Cd36* displayed low percentage expression in in vitro cultured PV-ADSCs, which suggested difficult retainment of these markers in present culture condition or selective expansion of cluster 2 cells. Future efforts including selective culture of cells from each cluster and separate determination of their contribution to vascular remodeling are needed.

Consistent with what has been reported for MSCs,^47,48^ considerable heterogeneity of PV-ADSCs has been observed in our study, which serves as direct evidence for stem cells from the perivascular (periaorta) adipose tissue. Although transcriptional network heterogeneity of stromal vascular fraction from axillary adipose tissue was reported,^49^ PVAT is more relevant to vascular function.^1^ With scRNA-seq, heterogeneous intracellular markers and functional modules have also been described in our study. Because of this substantial heterogeneity, markers that are used to identify MSC population by itself, such as *Tbx18*^37^, might only label a subpopulation. To overcome the laborious efforts to generate lineage tracing models for all the heterogeneously expressed markers used for MSC identification, more specific markers need to be discovered. In our study, *Tgfbr2* and *Anxa1* characterize cluster 2 PV-ADSCs specifically and bears the potential to identify this population homogenously in vivo. However, because of the limited number of primary PV-ADSCs, only a small number of cells were sequenced in our study. This is one limitation of the study. Sequencing of more primary cells would provide the possibility to discover better identity markers unique for MSC population or subpopulation. Another limitation of the study mainly lies in the lack of in vivo evidence for PV-ADSC contribution to vascular injury repair. Future investigations of in vivo PV-ADSC fate labeled by markers found by this study and future scRNA-seq data with larger cell number are needed.

Under disease settings such as atherosclerosis and vascular injury, adventitial stem cells migrate to the injury site and differentiate towards vascular lineages in response to the cytokine profile changes.^50,51^ Similar explorations of PV-ADSC responses in diseased status would provide novel therapeutic insights. Cell transplantation experiments of RFP-labeled PV-ADSCs in vein graft transplantation experiments confirmed their involvement in vascular remodeling, similar to adventitial stem cells. Preliminary result demonstrating the migration of PV-ADSCs towards chemoattractant SDF-1 suggests that cell migration is among the complex mechanisms through which PV-ADSCs participate in vascular injury repair and thus is a potentially interesting field. These findings together expand the pool of the resident vascular stem cell population consisting adventitial stem cells to include MSCs that reside in the PVAT. Lineage tracing studies with markers identified by scRNA-seq are in need to further assess the in vivo physiological and pathophysiological role of PV-ADSCs.

Regarding the differentiation mechanism, accumulating evidence has demonstrated the importance of metabolic reprogramming in stem cell differentiation.^11,44^ It was shown that increased glycolysis is associated with pluripotency and increased mitochondrial respiration is associated with differentiation in pluripotent stem cells.^52^ MSCs displayed suppressed glycolysis when driven to differentiation towards osteocytes.^53^ Our study characterized the metabolic profile change of PV-ADSCs during SMC differentiation and proposed the importance of lipid metabolism in regulating the differentiation process. MiR-378-3a bears potential in regulating SMC differentiation. Efforts are needed to further validate the function of miR-378-3a in vascular injury models and explore its therapeutic potential.

Evidence of the important role of the adipose-vascular crosstalk in various metabolic diseases has identified novel targets such as perivascular relaxing factors^54^ or secreted adipokines^55^ with significant therapeutic potential. By demonstrating the role of PV-ADSCs in vascular remodeling through metabolism-related regulation of SMC differentiation, our results support the need to further investigate the role of PV-ADSCs in vascular remodeling in various metabolic diseases such as atherosclerosis and diabetes mellitus. The process of PV-ADSC-involved vascular remodeling, which was not recognized before, might be targeted in future therapeutic development for metabolic diseases. Potential future exploration also includes the influence of adipokines secreted by PVAT on PV-ADSC differentiation and function, especially under disease conditions, given that preliminary results have shown the promoting effect of IGFBP-2 on SMC differentiation. Collectively, our study provides direct evidence of PV-ADSC heterogeneity through high-resolution characterization with scRNA-seq and suggests their participation in vascular remodeling through SMC differentiation driven by metabolic reprogramming which necessitate confirmation with further in vivo lineage tracing experiments. Taken together, the results imply the previously unappreciated identity and function of PV-ADSCs in vascular remodeling, unveil the mechanism for SMC differentiation from a metabolic perspective and provide insights for future studies to investigate the role of PV-ADSCs in various metabolic diseases.

## Acknowledgments

We acknowledge financial support from the Department of Health via the National Institute for Health Research (NIHR) comprehensive Biomedical Research Centre award to Guy’s & St Thomas’ NHS Foundation Trust in partnership with King’s College London and King’s College Hospital NHS Foundation Trust. Nuclear magnetic resonance (NMR) experiments were produced using the facilities of the Centre for Biomolecular Spectroscopy, King’s College London, acquired with a Multi-user Equipment Grant from the Wellcome Trust and an Infrastructure Grant from the British Heart Foundation. Breeding of miR-378a knockout mice was supported by the MAESTRO and OPUS grants of the Polish National Science Center (2012/06/A/NZ1/0004 and 2012/07/B/NZ1/02881) to J. Dulak.

## Sources of Funding

This work is supported by the British Heart Foundation (RG/14/6/31144).

## Disclosures

None.

## Supplementary Material
