# Supplementary material for: Single-Cell RNA-Sequencing and Metabolomics Analyses Reveal the Contribution of Perivascular Adipose Tissue Stem Cells to Vascular Remodeling
Source: Arterioscler Thromb Vasc Biol. 2019 Jul 25;39(10):2049–66. doi: 10.1161/ATVBAHA.119.312732 (PMC6766361; doi:10.1161/ATVBAHA.119.312732)
Supplement: Supplementary file 1 [file atv-39-2049-s001.pdf]

Major Resources Tables

Animals (in vivo studies)

Species	Vendor or Source	Background Strain	Sex
Mouse (wt)	Jackson Laboratory	C57BL/6	Male
Mouse (129SvEv/C57BL/6/mir-378-KO)	Dr. Eric Olson (University Texas Southwestern Medical Centre, Dallas, USA)	C57Bl/10ScSn-Dmdmdx/J	Male and Female

Antibodies: WB: Western Blot, IF: immunofluorescent staining, FC: flow cytometry

Target antigen	Vendor or Source	Catalog #	Working dilution
anti-CD45-APC	BD Biosciences	561018	FC: 0.5 µg/ml
anti-CD29-PE	BD Biosciences	562801	FC: 0.5 µg/ml
anti-Sca-1-PE-Cy7	Biolegend	108113	FC: 0.5 µg/ml
anti-CD31-PerCP	Biolegend	201419	FC: 0.5 µg/ml
anti-CDH5-Alexa Fluor 647	BD Biosciences	562242	FC: 0.5 µg/ml
anti-PDGFRa-APC	eBioscience	17-1401-81	FC: 0.5 µg/ml
anti-CD117-PE	Biolegend	105807	FC: 0.5 µg/ml
anti-CD34-APC	Biolegend	128611	FC: 0.5 µg/ml
anti-CD44-PerCP	Biolegend	103035	FC: 0.5 µg/ml
anti-CD11b-PE	Biolegend	101205	FC: 0.5 µg/ml
anti-FABP4	Abcam	ab92501	IF: 1 µg/ml
anti-OPN	R&D systems	AF808	IF: 1 µg/ml
anti-PLIN1	Abcam	ab3526	IF: 5 µg/ml
anti-CD31-AlexaFluor 647	Biolegend	102516	IF: 2.5 µg/ml
anti-Sca-1	Abcam	ab51317	IF: 5 µg/ml
anti-CD29	R&D Systems	AF2405-SP	IF: 1 µg/ml
anti-CNN1	Abcam	ab46794	WB: 0.05 µg/ml<br>IF: 0.2 µg/ml
anti-TAGLN	Abcam	ab14106	WB: 0.5 µg/ml<br>IF: 2 µg/ml
anti-ACTA2	Sigma	2547-100	WB: $2X10^{-6}$ M
anti-ACTA2	Sigma	C6198–2ML	IF: $2X10^{-5}$ M
anti-ACTA2	Abcam	ab184675	IF: 2.5 µg/ml
anti-TGFb1	Abcam	ab92486	WB: 0.5 µg/ml<br>IF: 5 µg/ml
anti-RFP	Rockland	600-401-379	IF: 5 µg/ml

Q-PCR primers

**Gene Symbol**	**Species**	**Sequence (5’→3’)**
Acta2	Mouse	F: 5’-TCAGGGAGTAATGGTTGGAATG-3’<br>R: 5’-GGTGATGATGCCGTGTTCTA-3’
Bglap	Mouse	F: 5’-GCAATAAGGTAGTGAACAGACTCC-3’<br>R: 5’-GTTTGTAGGCGGTCTTCAAGC-3’
Cebpa	Mouse	F: 5’-ATACTCAAAACTCGCTCCTTTTCC-3’

		R: 5’-GGATCTCAGCTTCCTGTATCTTCC-3’
Cebpb	Mouse	F: 5’-GTAATCACTTAAAGATGTTCCTGCGG-3’<br>R: 5’-ACTTTAATGCTCGAAACGGAAAAGG-3’
Cebpd	Mouse	F: 5’-CCTTCTACGAGCCAGGCAGG-3’<br>R: 5’-GTCAATGTAGGCGCTGAAGTCG-3’
Cnn1	Mouse	F: 5’-GGTCCTGCCTACGGCTTGTC-3’<br>R: 5’-TCGCAAAGAATGATCCCGTC-3’
Cpt1a	Mouse	F: 5’-CTCCGCTCGCTCATTCC-3’<br>R: 5’-TGCCATTCTTGAATCGGATG-3’
Cpt1b	Mouse	F: 5’-ATGGTGGGCAACTAACTATGT-3’<br>R: 5’-AGTTTGCGGCGATACATGA-3’
Crat	Mouse	F: 5’-AAGAAAATGGAGAACTGGCTGTCC-3’<br>R: 5’-ACCCTCGATGAGTTTGGCAGC-3’
Fabp4	Mouse	F: 5’-TGTCTCCAGTGAAAACTTCG-3’<br>R: 5’-TGACCAAATCCCCATTTACG-3’
Ppara	Mouse	F: 5’-TACTGCCGTTTTCACAAGTGC-3’<br>R: 5’-AGGTCGTGTTCACAGGTAAGA-3’
Ppard	Mouse	F: 5’-GAAGAGGAGAAAGAGGAAGTGGC-3’<br>R: 5’-GAGGAAGGGGAGGAATTCTGG-3’
Pparg	Mouse	F: 5’-GTGATCTTAACTGCCGGATCCAC-3’<br>R: 5’-CTCTGGGTTCAGCTGGTCGATA-3’
Prdm16	Mouse	F: 5’-AAGAAAATGGAGAACTGGCTGTCC-3’<br>R: 5’-ACCCTCGATGAGTTTGGCAGC-3’
Runx2	Mouse	F: 5’-ACAGTCTTCACAAATCCTCC-3’<br>R: 5’- GACACCTACTCTCATACTGG-3’
Tagln	Mouse	F: 5’-GATATGGCAGCAGTGCAGAG-3’<br>R: 5’-AGTTGGCTGTCTGTGAAGTC-3’
Tgfb1	Mouse	F: 5’-CCACCTGCAAGACCATCGAC-3’<br>R: 5’-CTGGCGAGCCTTAGTTTGGAC-3’
Ucp1	Mouse	F: 5’-CCATGTACACCAAGGAAGGACC-3’<br>R: 5’-CAAGTTGCTTATGTGGTACAATCC-3’
