# Supplementary figures and images for: Single-Cell RNA-Sequencing and Metabolomics Analyses Reveal the Contribution of Perivascular Adipose Tissue Stem Cells to Vascular Remodeling
Source: Arterioscler Thromb Vasc Biol. 2019 Jul 25;39(10):2049–66. doi: 10.1161/ATVBAHA.119.312732 (PMC6766361; doi:10.1161/ATVBAHA.119.312732)

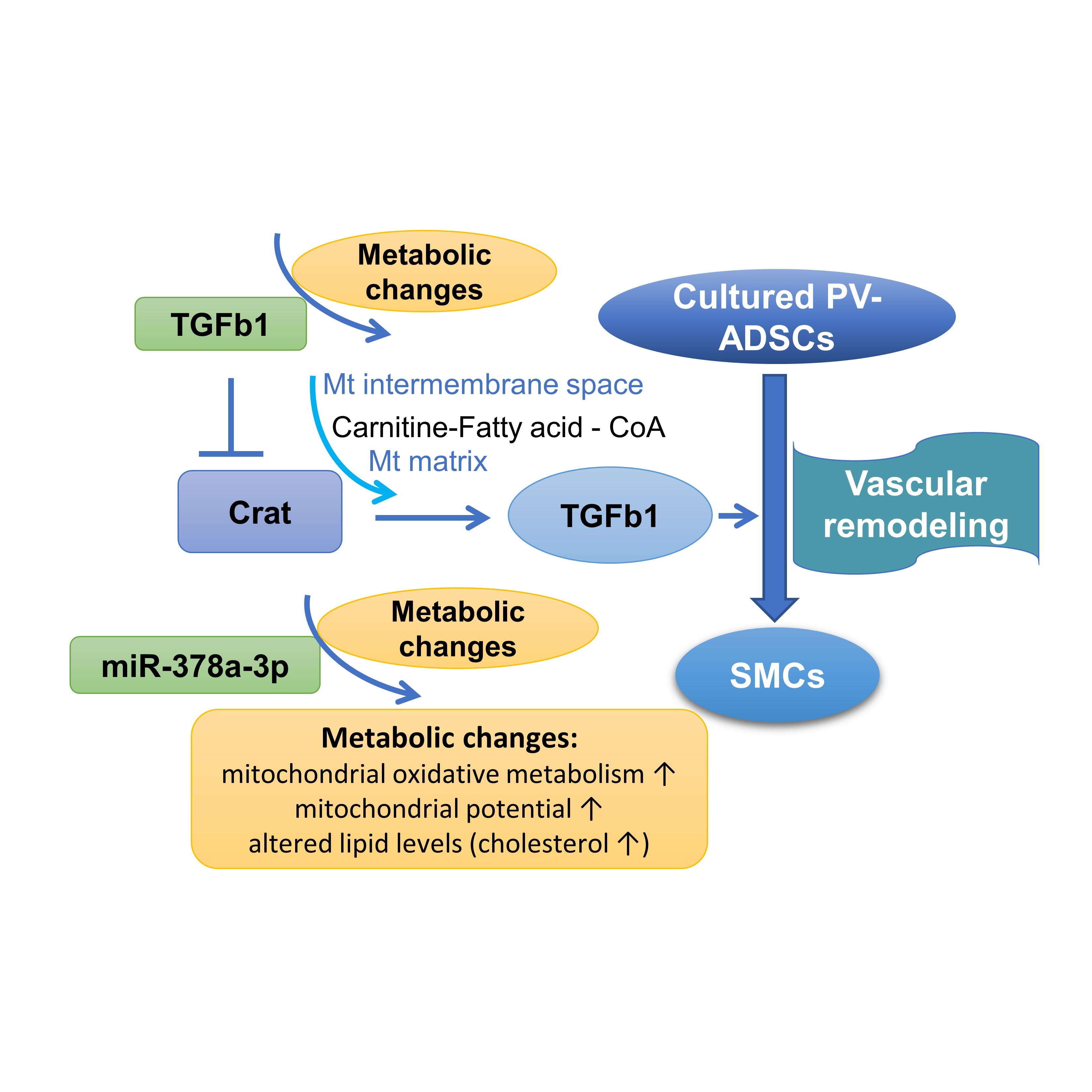

Supplement: Supplementary file 2 [file atv-39-2049-s002.jpg]
