# Supplementary material for: Single-Cell RNA-Sequencing and Metabolomics Analyses Reveal the Contribution of Perivascular Adipose Tissue Stem Cells to Vascular Remodeling
Source: Arterioscler Thromb Vasc Biol. 2019 Jul 25;39(10):2049–66. doi: 10.1161/ATVBAHA.119.312732 (PMC6766361; doi:10.1161/ATVBAHA.119.312732)
Supplement: Supplementary file 3 [file atv-39-2049-s003.pdf]

Single-cell RNA-sequencing and Metabolomics Analyses Reveal the Contribution of Perivascular Adipose Tissue Stem Cells to Vascular Remodeling

Online-only Data Supplement

- **Supplemental Materials and Methods**
- **Supplemental Figures**
- **Supplemental Tables**

Supplemental Materials and Methods

Adipogenic and osteogenic differentiation of PV-ADSCs

Cultured PV-ADSCs were seeded on gelatin coated flasks and then changed to adipogenic or osteogenic differentiation medium and maintained for 2 weeks before being harvested for Q-PCR or subjected to immunofluorescent staining. Differentiation medium was composed of StemXVivo Osteogenic/Adipogenic Base Media (R&D systems, CCM007) with either StemXVivo Adipogenic Supplement (R&D systems, CCM011) or StemXVivo Osteogenic Supplement (R&D systems, CCM009). Immunofluorescent staining of FABP4 (Abcam, ab92501) and OPN (R&D systems, AF808) was utilized to confirm the adipogenic and osteogenic differentiation respectively.

RNA (including miRNA) extraction, RT-PCR and quantitative real-time PCR (Q-PCR)

Total RNA was extracted using the RNeasy mini kit (Qiagen, 74106) following the kit instructions. Reverse transcription of RNA was performed with the QuantiTect Reverse Transcription Kit (Qiagen, 205314). 1000 ng of total RNA was used for each reaction of reverse transcription. Gene expression level in cells was detected by Q-PCR using Eppendorf Mastercycler ep realplex in duplicates. Fold change of gene of interest was calculated by the threshold cycle value difference against internal control GAPDH. Extraction of microRNA was performed with miRNeasy mini kit (Qiagen, 217004) following the kit instructions. Reverse transcription of miRNA was performed with MicroRNA Reverse Transcription Kit (Life Tech, 4366597). Specific TaqMan microRNA assay and TaqMan Master Mix (Life Tech, 4440040) were used to assess the expression of microRNAs. All samples were run in duplicates and standardized to U6.

Western blot

10-50 µg of the protein was mixed with 1xSDS loading buffer and then boiled at 95°C for 10 minutes before being loaded to the NuPage 4-12% Bis Tris gel immersed in NuPage MOPS SDS running buffer in a XCell SureLock Mini-Cell (Life Tech, NP0335BOX). Protein ladder (Bio-rad, 161-0374) was loaded simultaneously with the samples and gel was run at 160V for about 75 minutes until the marker with the lowest molecular weight was at the bottom of the gel. Protein was transferred from the gel to the membrane (VWR, 732-3031) in 1X transfer buffer (Life Tech, NP-0006-1) for 45 minutes. The membrane was then blocked with 5% milk in PBS-Tween and incubated with primary antibody diluted in 5% milk at room temperature for 2 hours or at 4°C overnight. Following primary antibodies were used: CNN1 (Abcam, ab46794), TAGLN (Abcam, ab14106), ACTA2 (Sigma, 2547-100), and TGFb1 (Abcam, ab92486). Secondary antibodies were purchased from Dako (Rabbit anti-goat, P0449; Rabbit anti-mouse, P0260; Swine anti-rabbit, P0217). Further 3 washes were performed before addition of ECL detection solutions (Fisher Scientific, RPN2106). After incubation with the detection solution for 2 minutes, exposure of the films (Fisher Scientific, 28-9068-37) was carried out with the Compact X4 (Xograph Imaging System).

Immunostaining

Mouse aortas with peri-aorta adipose tissue were snap frozen in liquid nitrogen and stored in -80 °C. Cells seeded on 8-well chamber slides (Millipore, PEZGS0816) coated with gelatin were fixed with 4% paraformaldehyde for 15 minutes at room temperature. Section were fixed with acetone and then blocked for 1 h with 10% donkey serum in PBS with 0.1% Tween-20. Specimens were stained overnight with following antibodies: anti-PLIN1 (Abcam, ab3526), anti-CD31-AlexaFluor 647 (BD Pharmingen, clone MEC13.3), anti-Sca-1 (Abcam, ab51317), anti-CD29 (R&D Systems, AF2405-SP), anti-CNN1 (Abcam, ab46794), anti-TAGLN (Abcam, ab14106), anti-ACTA2 (Sigma, C6198–2ML or Abcam, ab184675), anti-TGFb1 (Abcam, ab92486), anti-RFP (Rockland, 600-401-379). and DAPI. Images were taken with Axio Imager M2 microscope or Leica SP5 confocal microscope and analyzed with LAS AS software (Leica).

Histological analysis

Tissues including subcutaneous *Matrigel* plugs and vein grafts from experimental mice were fixed with 4% formalin overnight at 4°C prior to a machine-based dehydration. The dehydrated

samples were embedded in paraffin and subsequently cut into 7 µm sections. H&E staining was performed using a standard protocol with Hematoxylin and Eosin for morphology analysis.

Glucose uptake assay

For the analysis of glucose uptake cells were incubated for 30 minutes in standard culture conditions with medium containing 10 µmol/L 2-(N-(7-nitrobenz-2-oxa-1,3-diazol-4-yl) amino)-2-deoxyglucose (2-NBDG, Thermo Scientific, N13195). Cells were then detached with trypsin and analyzed with BD Accuri C6 flow cytometer.

Mitochondrial potential measurement

Tetramethylrhodamine, Methyl Ester, Perchlorate (TMRM) was used to measure the mitochondrial potential. PV-ADSCs were treated as indicated and at the day of assay, staining medium (αMEM with 10% FBS and 100 mmol/L TMRM) was added to the cells. After incubation for 30 mins, the cells were detached with trypsin and subjected to fluorescent detection with BD LSR Fortessa flow cytometer.

Trans-well Assay

Migration assays were performed using transwell inserts with 8.0 µm pore membrane filters (Corning, 3422). Cultured PV-ADSCs (10^5 cells/ml serum-free medium) were seeded into the upper chamber of the trans-well. The bottom chamber contained serum-free medium with control or murine SDF-1 (Peprotech) at different concentrations. Serum free medium served as negative control. After 16-h incubation, non-migrating cells on the upper side of the filters were carefully washed and removed using a swab. The migrated cells on the lower surface of transwell filter were fixed in 4% PFA for 10 min and then stained with 1% crystal violet (Sigma, HT90132) for 15 min. Images were acquired using Nikon Eclipse TS100 microscope. Cells were counted in 5 random fields under the microscope for statistical analysis.

Cytokine profile of PV-ADSCs

Various cytokines in cell lysates of SC-ADSCs and PV-ADSCs were detected with Proteome Profiler Mouse Angiogenesis Array Kit (R&D systems, ARY015) following the manual.

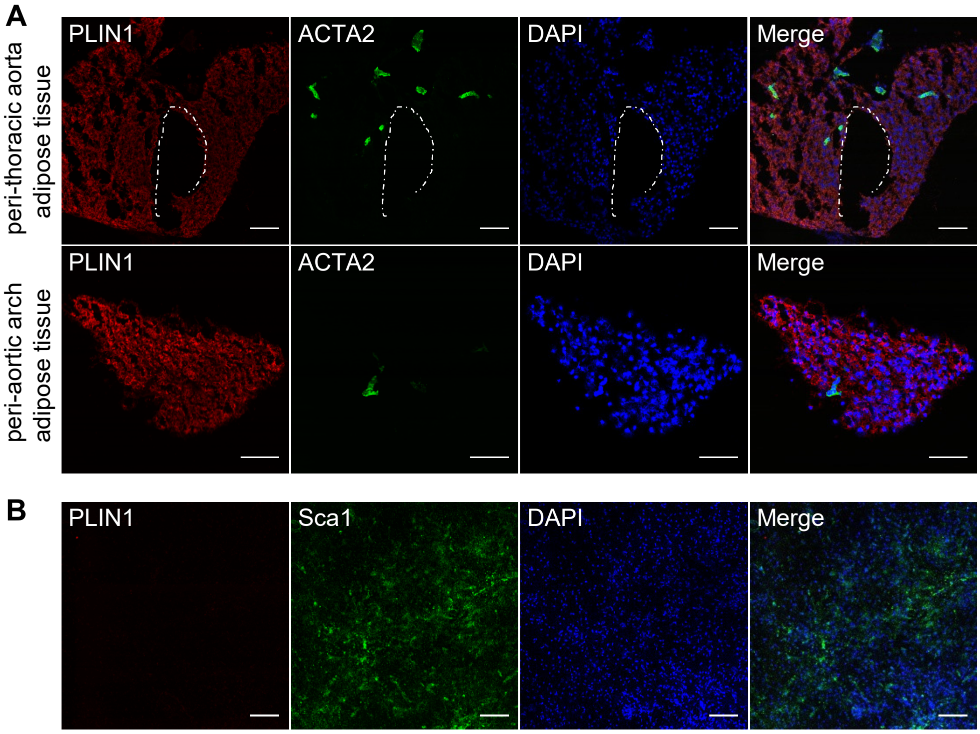

Supplemental Figure I. Isolation of peri-aorta adipose tissue from the adventitia. A, Immunofluorescent staining of adipose tissue section surrounding the thoracic aorta and aortic arch with adipocyte marker PLIN1 and smooth muscle marker ACTA2. Dashed line indicates the side adjacent to aorta. **B**, *En face* immunofluorescent staining of remaining thoracic aorta adventitia after isolation of peri-aorta adipose tissue. Scale bar, 100 μm. DAPI, 4′,6-diamidino-2-phenylindole.

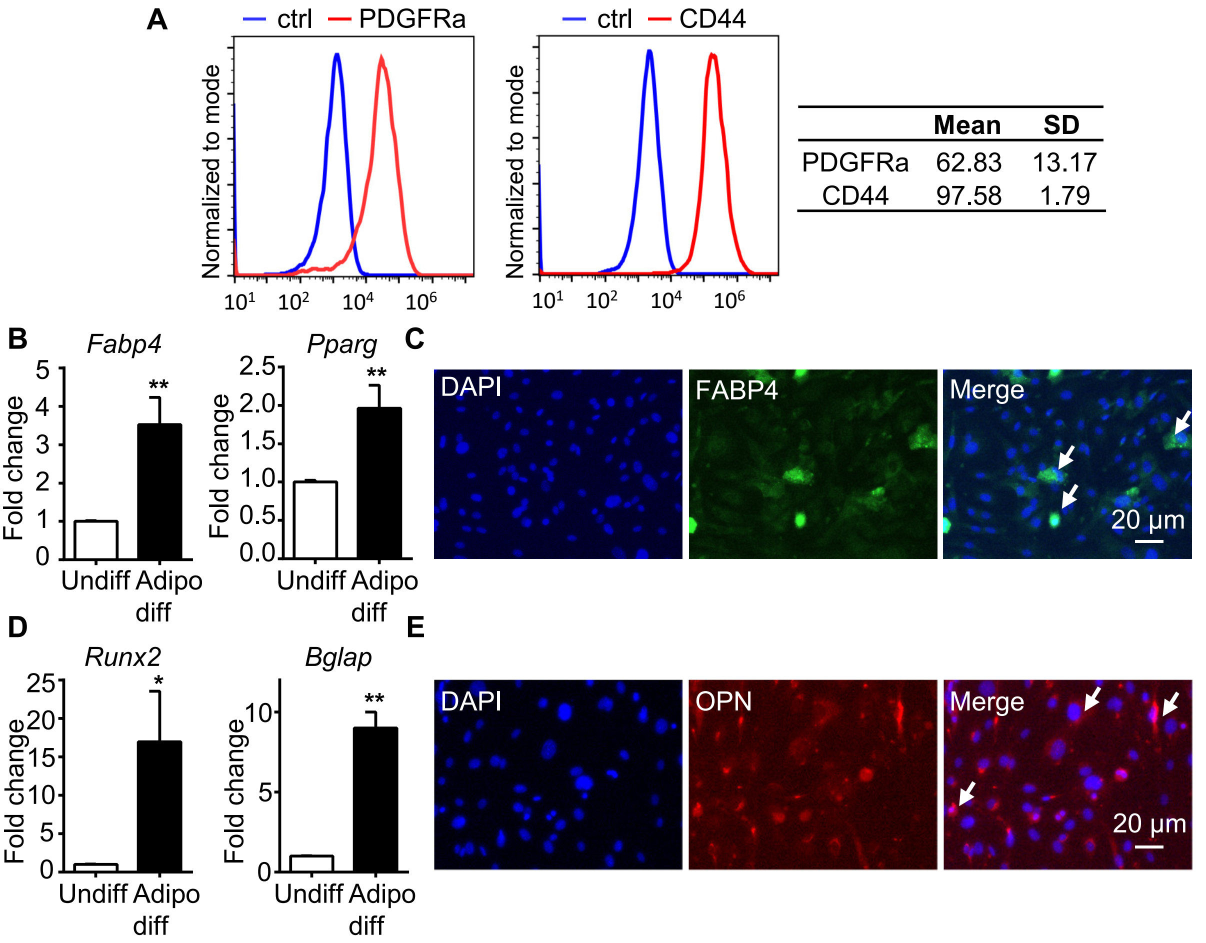

Supplemental Figure II. Characterization of PV-ADSCs. A, Flow cytometry analysis and statistics of *in vitro* cultured PV-ADSCs. **B** Q-PCR analysis of *Fabp4* and *Pparg* of PV-ADSCs derived adipocytes in comparison with undifferentiated cells (n=3). **C**, Immunofluorescent staining of FABP4 (green) of differentiated PV-ADSCs towards adipocytes. Arrows indicate FABP4 positive adipocytes. **D**, Q-PCR analysis of *Runx2* and *Bglap* of PV-ADSCs derived osteocytes in comparison with undifferentiated cells (n = 3). **E**, Immunofluorescent staining of OPN (red) of differentiated PV-ADSCs towards osteocytes (n=3). Arrows indicate OPN positive osteocytes. Data are presented as mean±SD. **P*<0.05 and ***P*<0.01. DAPI, 4′,6-diamidino-2-phenylindole; and OPN, osteopontin.

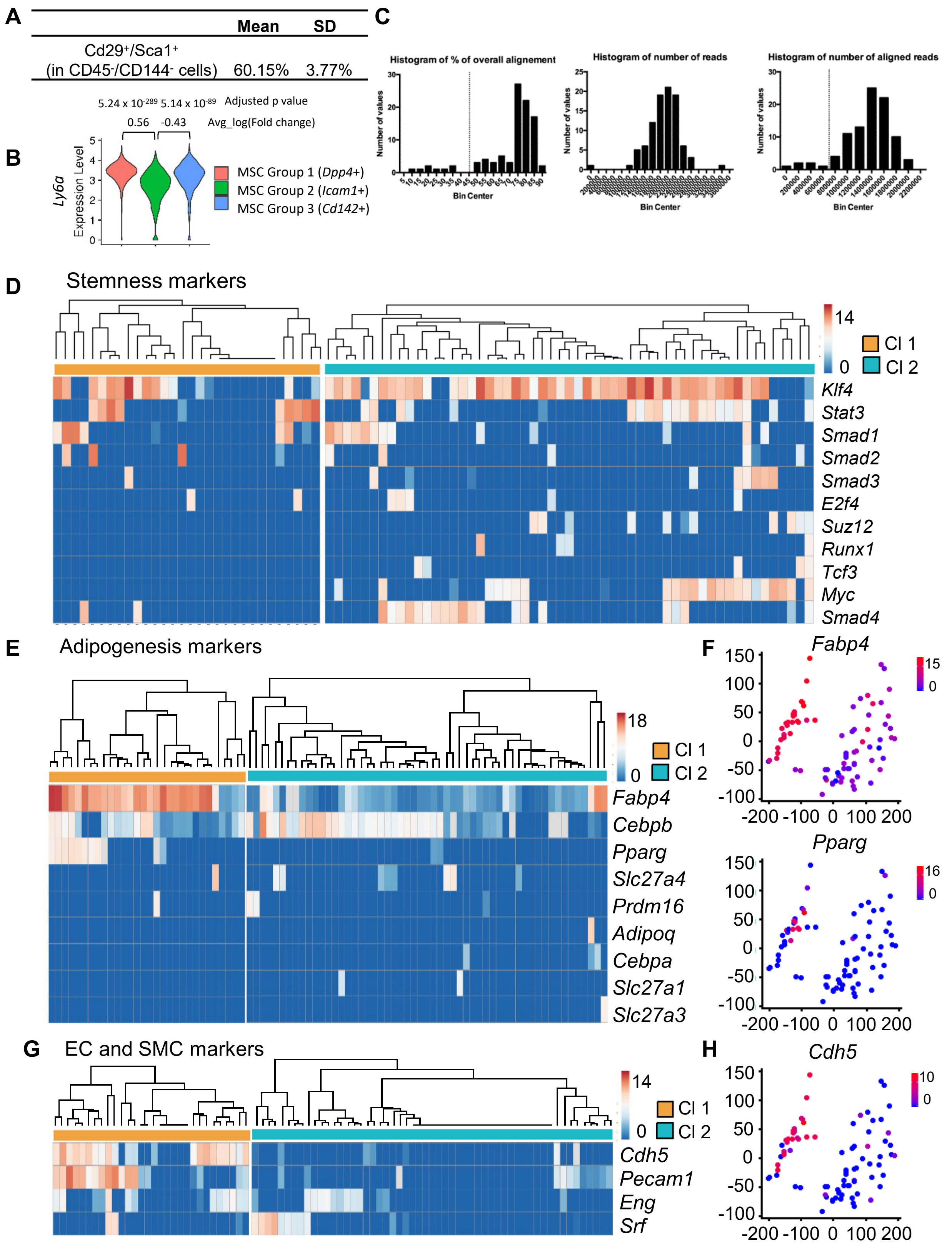

Supplemental Figure III. Expression of selected markers in primary PV-ADSCs. A, Percentage of CD29^{+}/Sca1^{+} cells in non-immune non-endothelial cells of freshly isolated peri-aorta adipose tissue. **B**, Expression of *Ly6a* in the three main mesenchymal cell groups identified by Merrick *et al* (GSM3717978). **C**, Histogram showing the overall alignment, number of reads and number of aligned reads of primary PV-ADSC scRNA-seq data. **D**, Heatmap of stemness marker expression in primary PV-ADSCs. **E**, Single-cell level expression of the adipogenesis markers in primary PV-ADSCs. **F**, Expression level of *Fabp4* and *Pparg* in primary PV-ADSCs. **G**, Single-cell level expression of endothelial markers (*Cdh5*, *Pecam1* and *Eng*) and SMC markers (*Srf*) in primary PV-ADSCs was shown as heatmap. **H**, Representative graph showed the expression of *Cdh5* in primary PV-ADSCs. Cl 1, cluster 1; Cl 2, cluster 2; EC, endothelial cell; MSC, mesenchymal stem cell; and SMC, smooth muscle cell. Color scale: log2(gene counts).

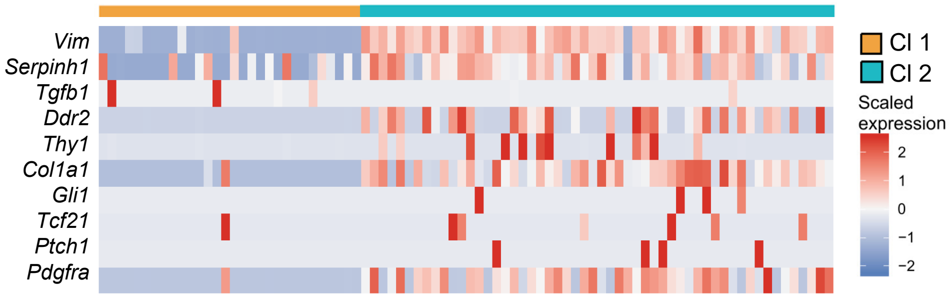

Supplemental Figure IV. Expression of fibroblast markers in primary PV-ADSCs. Heatmap of fibroblast marker expression in primary PV-ADSCs. Cl 1, cluster 1; and Cl 2, cluster 2.

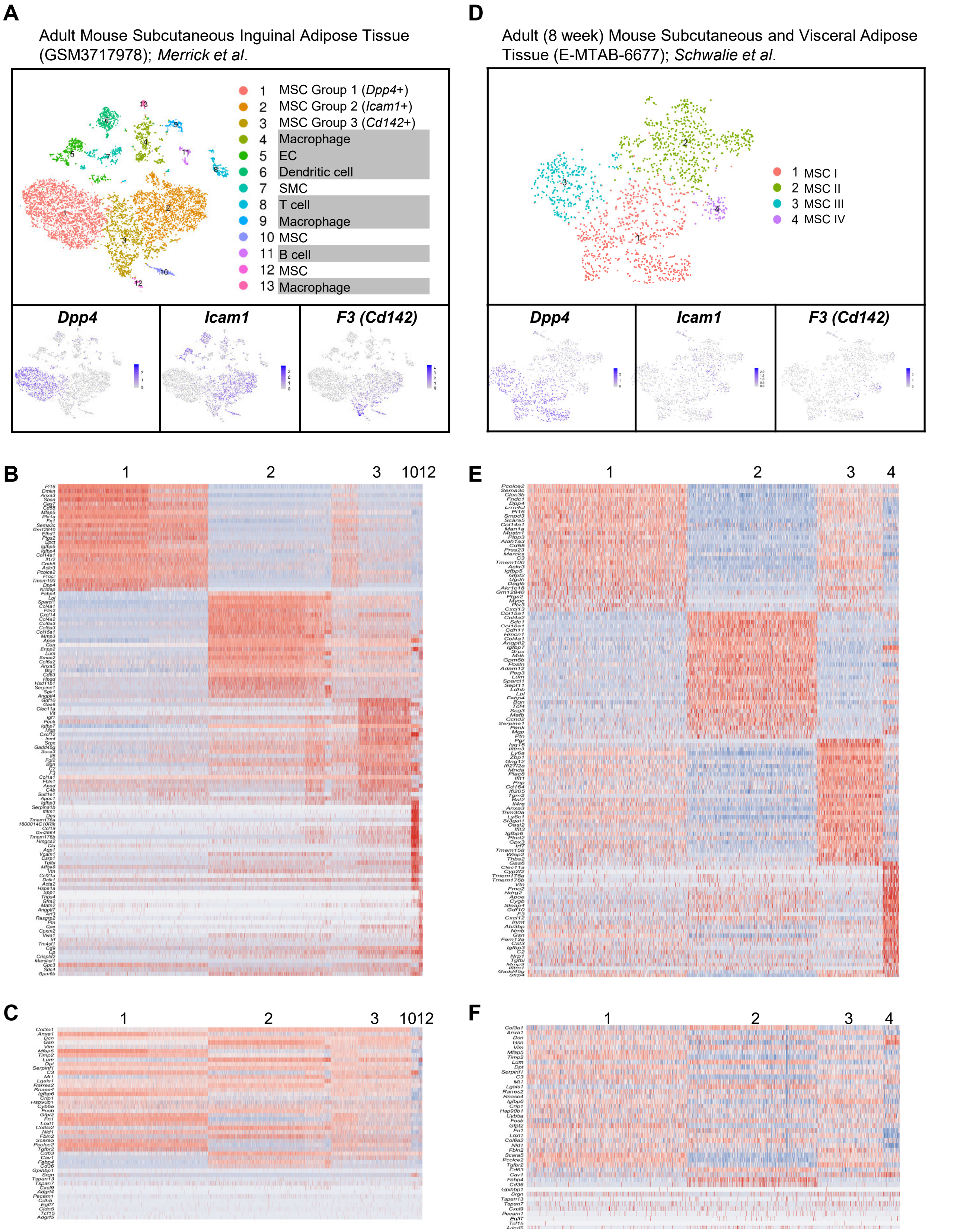

Supplemental Figure V. Heterogeneity of subcutaneous adipose tissue stromal vascular fraction in published datasets. A-C, Analysis of adult subcutaneous adipose tissue cells (GSM3717978). **D-F**, Analysis of 8-week-old subcutaneous and visceral adipose tissue stromal vascular fraction (E-MTAB-6677). **A** and **D**, t-SNE plot showed the distribution of annotated clusters. **B** and **E**, Heatmap of cluster-specific markers in mesenchymal populations. **C** and **F**, Heatmap of genes shown in Figure 2D.

A

Parameter	Cultured PV-ADSCs
Estimated Number of Cells	12,158
Mean Reads per Cell	27,091
Median Genes per Cell	2,602
Valid Barcodes	92.2%
Sequencing Saturation	30.6%
Q30 Bases in Barcode	97.0%
Q30 Bases in RNA Read	90.4%
Q30 Bases in UMI	96.9%
Reads Mapped Confidently to Transcriptome	63.4%
Reads Mapped Confidently to Exonic Regions	66.6%
Reads Mapped Confidently to Intronic Regions	12.4%
Reads Mapped Confidently to Intergenic Regions	3.1%

B

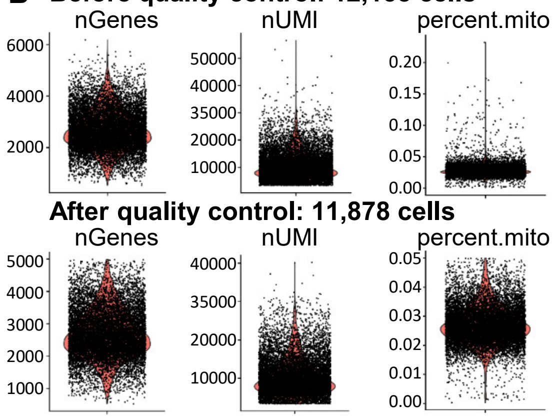

C

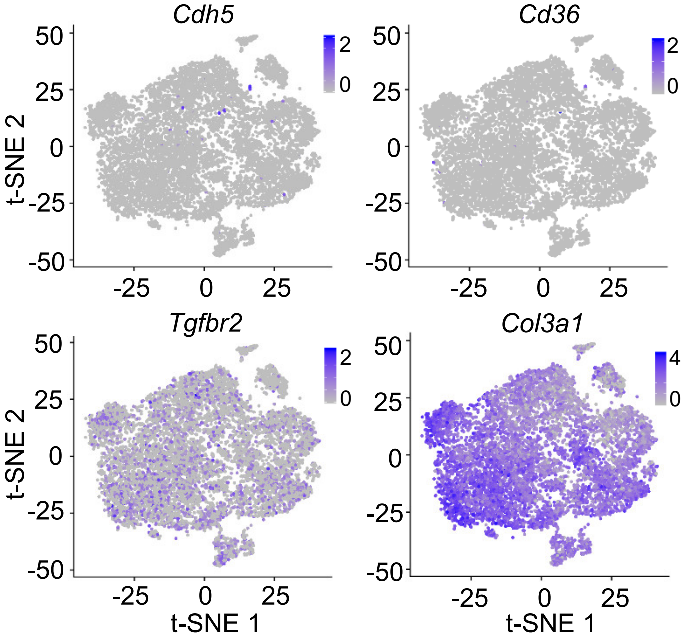

D

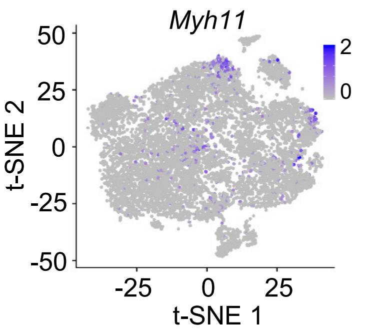

E

	Number	Percentage (%)
Total	11878	
Cdh5+	57	0.48
Cd36+	27	0.23
Tgfbr2+	3476	29.26
Col3a1+	11140	93.79
Myh11+	728	6.13

F

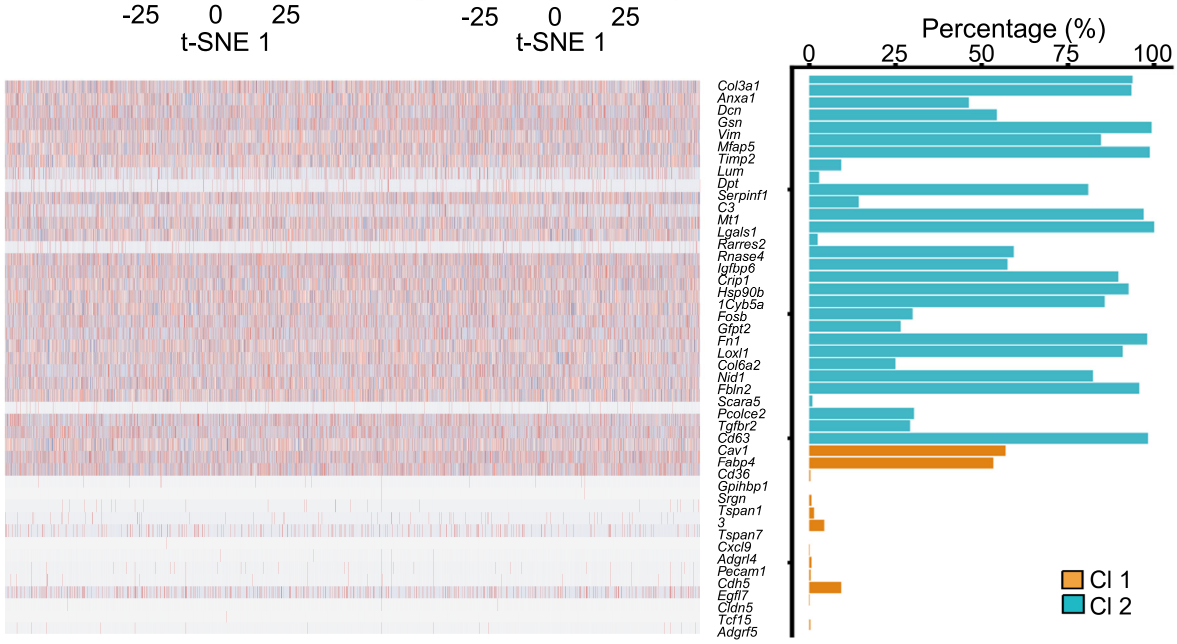

Supplemental Figure VI. Quality of single-cell RNA sequencing data and expression of cluster 1 and cluster 2 marker genes in cultured PV-ADSCs. A, Overall quality of scRNA-seq data. **B**, Violin plots of basic features of sequencing data. **C**, Expression of markers (*Cd36, Cdh5, Tfgbr2,* and *Col3a1*) was visualized in t-SNE graph. **D**, Expression of mature SMC marker *Myh11* was visualized in t-SNE graph. **E**, Percentage of cells expressing specific markers in cultured PV-ADSCs. **F**, Heatmap and corresponding percentage of cluster 1 and cluster 2 markers for primary PV-ADSCs in *in vitro* cultured PV-ADSCs. Color scale: scaled expression. nUMI, number of unique moledular identifier; nGene, number of genes; percent.mito, percentage of mitochondrial genes and t-SNE, t-distributed stochastic nearest neighbor embedding.

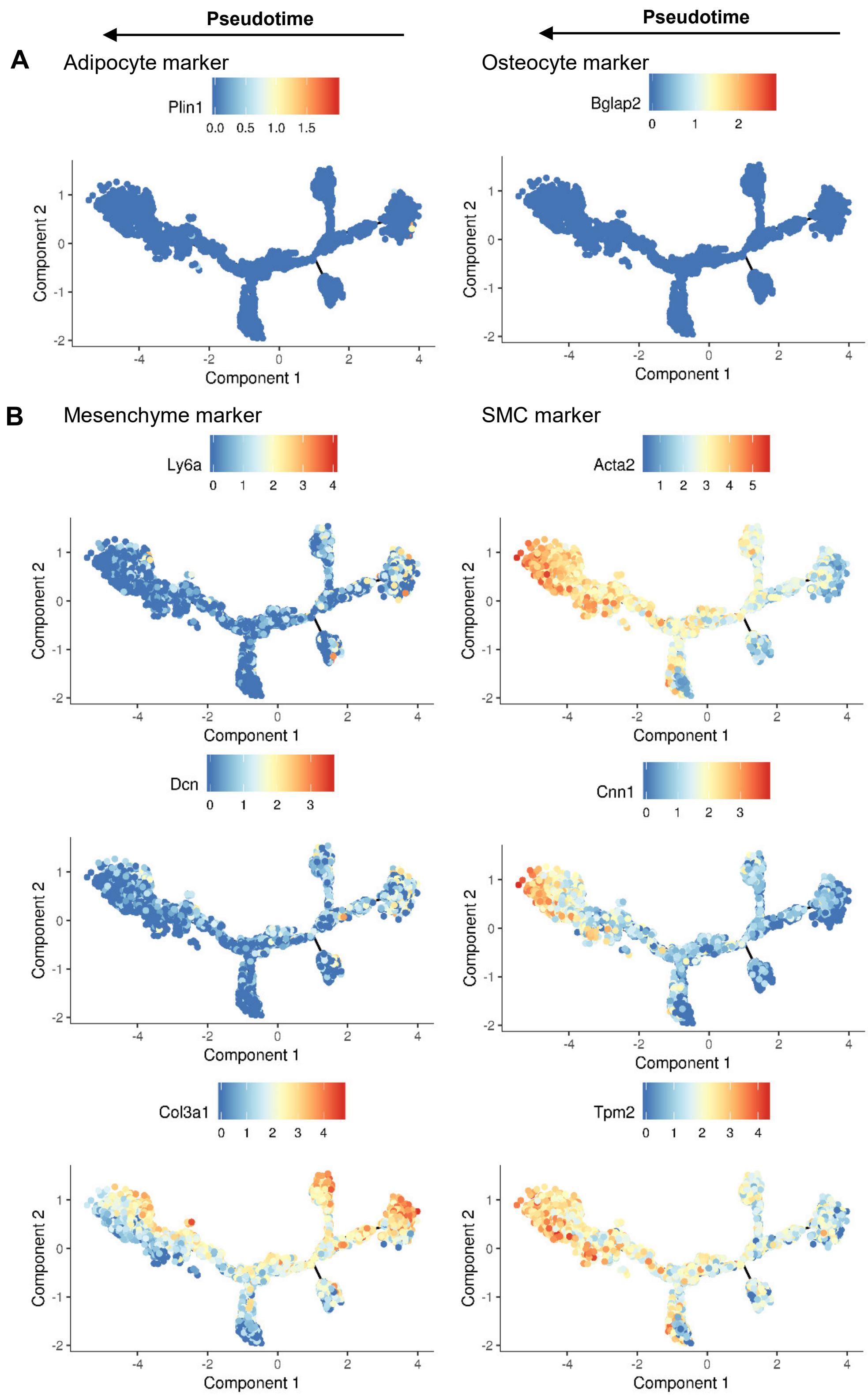

Supplemental Figure VII. Expression of selected markers along the trajectory. A, Expression of adipocyte marker *Plin1* and osteocyte marker *Bglap2* along the trajectory. **B,** Expression of mesenchyme and SMC markers along the trajectory. SMC indicates smooth muscle cell.

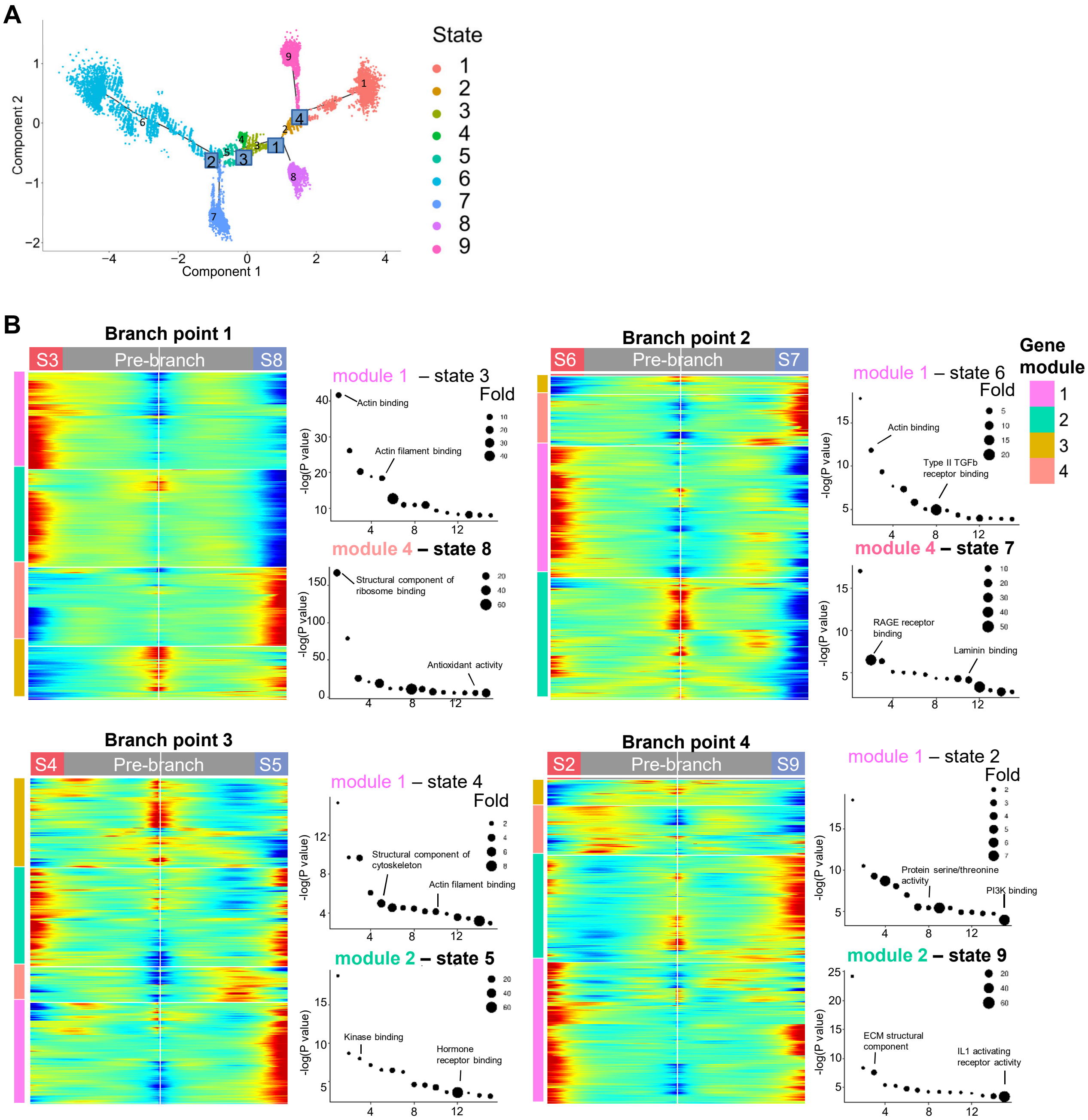

Supplemental Figure VIII. Branch analysis of pseudotime trajectory. A, Different states of cells along the pseudotime trajectory. Branch point is shown in framed text in the trajectory map. **B**, Heatmap of the top 1000 (by q value) significantly changed genes in four gene modules. Gene ontology (molecular function) analysis of the upregulated module of genes in the two states after branch point are analyzed and shown on the right side of the heatmap. Top panel by each heap map indicates gene ontology analysis with genes upregulated in cell states with a smaller number after the branch point. Lower panel by each heap map indicates gene ontology analysis with genes upregulated in cell states with a bigger number after the branch point. S indicates state.

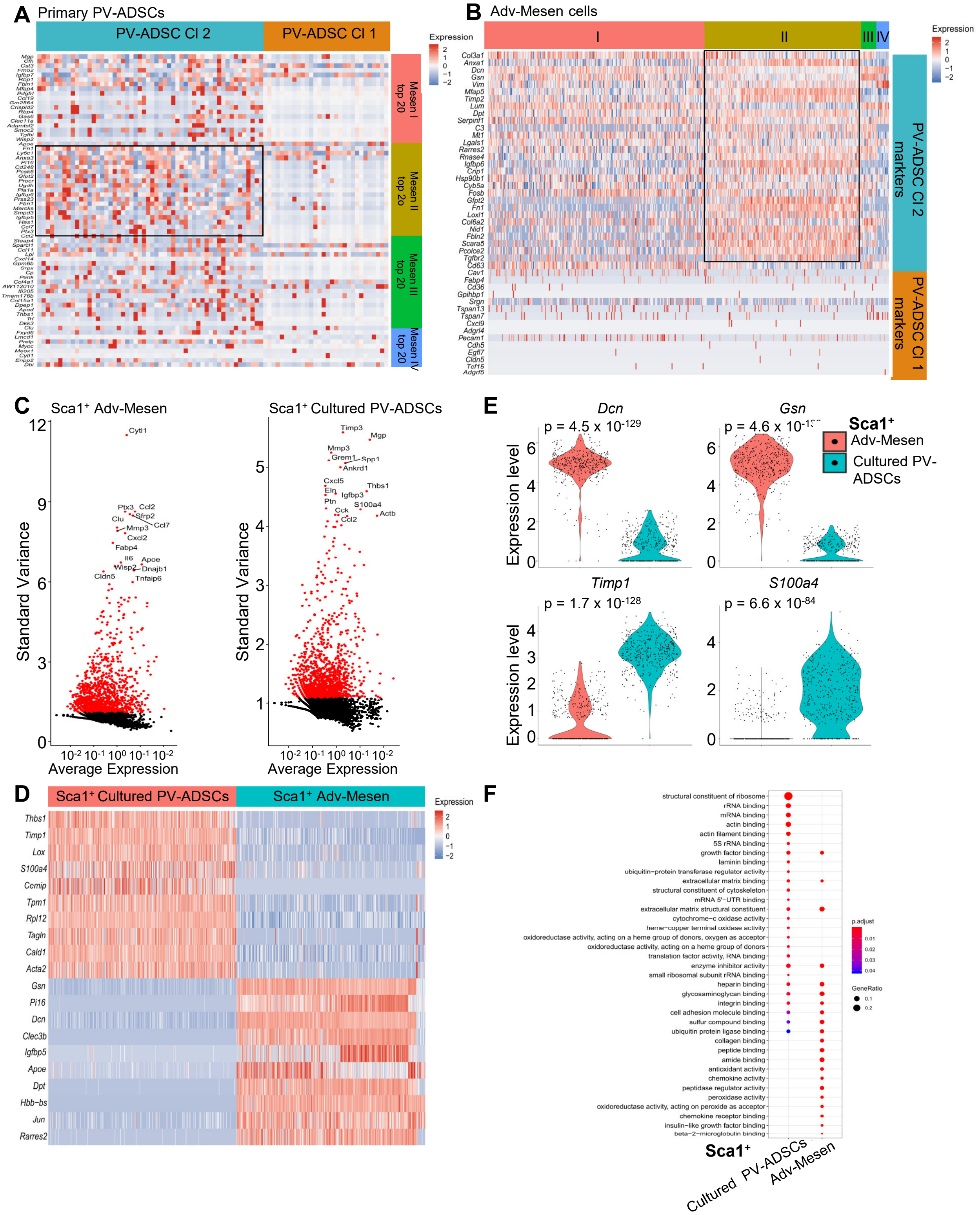

Supplemental Figure IX. Comparison of adventitial non-immune mesenchyme cells and PV-ADSCs. A, Expression heatmap of top 20 cluster-defining markers (Adv-Mesen cells, Gu *et al.*) in primary PV-ADSCs. Markers not found in PV-ADSCs were not shown. **B**, Expression heatmap of cluster-defining markers (primary PV-ADSCs, Fig. 2D) in Adv-Mesen cells. **C**-**F**, Analysis of Sca1⁺ Adv-Mesen cells (normalized Sca1 expression > 1, in total 400 cells) and cultured PV-ADSCs (randomly selected 400 cells with normalized Sca1 expression > 1). **C**, Variable genes. **D**, Heatmap of top 10 [by average log (fold change)] differentially expressed genes. **E**, Violin plot of selected markers. **F**, Selected GO terms enriched in Sca1⁺ cultured PV-ADSCs and Adv-Mesen cells. Adv-Mesen, adventitial mesen I-IV clusters; and PV-ADSC, peri-aorta adipose tissue-derived mesenchymal stem cells.

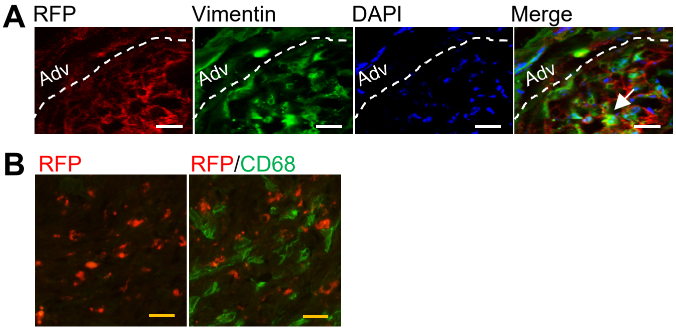

Supplemental Figure X. PV-ADSCs differentiate towards SMCs *in vivo.* A, Vein grafts harvested one week after the transplantation were stained with RFP (red) and fibroblast marker Vimentin (green) (n=4). Arrows indicate cells positive for both markers. Dashed line indicates boundary of adventitia. Scale bar, 20 μm. **B**, Neointima of vein grafts harvested one week after the transplantation were stained with RFP and CD68 (n=4). Arrows indicate cells positive for both markers. Scale bar, 50 μm. Adv indicates adventitia.

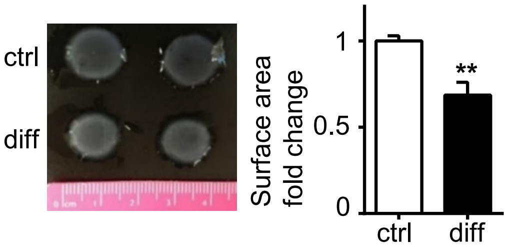

Supplemental Figure XI. Contractility of PV-ADSC-derived SMCs. Representative image of collagen gel contraction assay and analysis showed the contractility of differentiated PV-ADSCs (n=3). Data are presented as mean±SD. ***P*<0.01.

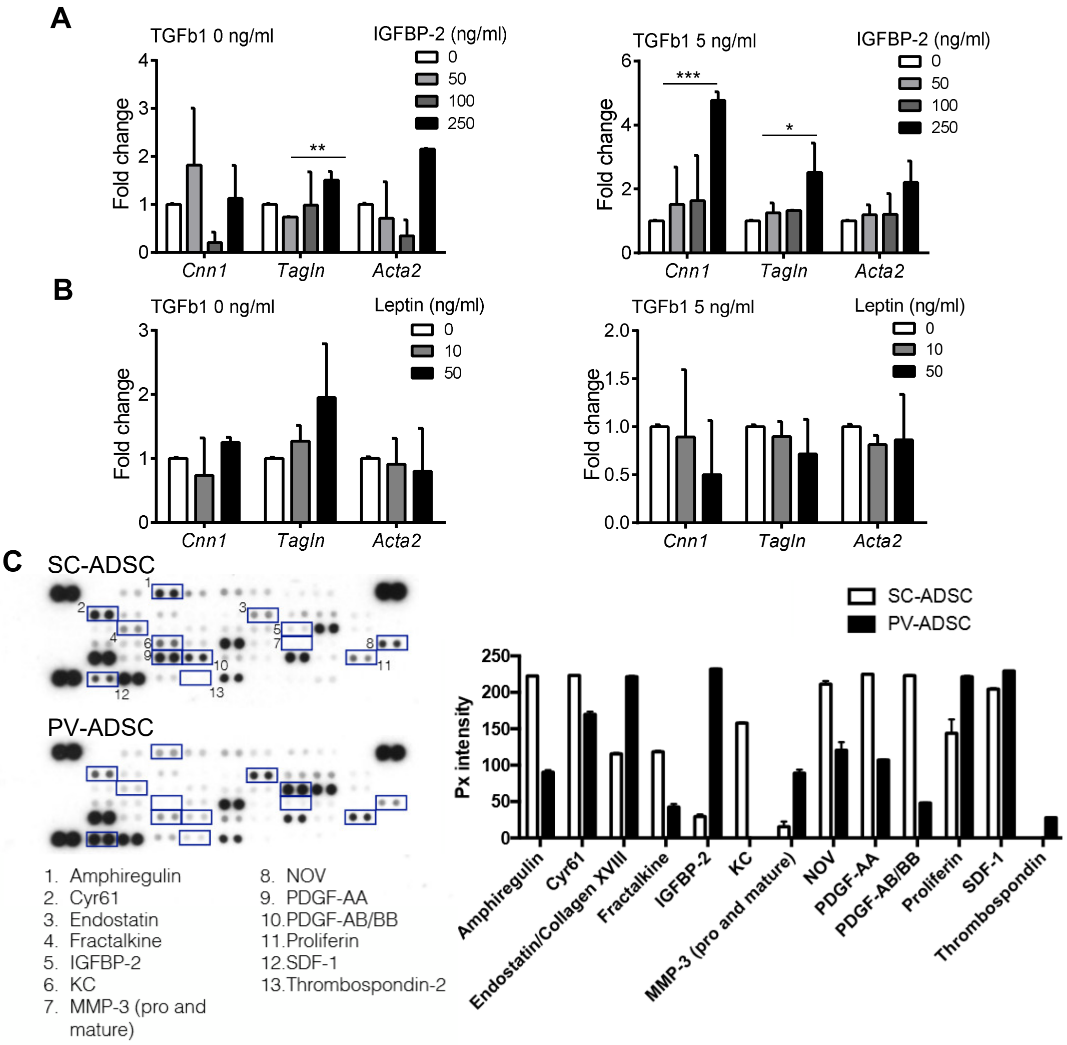

Supplemental Figure XII. PV-ADSC and adipokines. A, Gene expression of SMC markers in PV-ADSCs treated with leptin at indicated concentrations for 2 days in medium with or without TGFb1. **B**, Gene expression of SMC markers in PV-ADSCs treated with IGFBP2 at indicated concentrations for 2 days in medium with or without TGFb1. **C**, Protein level expression of various cytokines in PV-ADSCs and SC-ADSCs and statistic analysis of differentially expressed cytokines in the two cell types. Data are presented as mean±SD. **P*<0.05, ***P*<0.01. PV-ADSC indicates perivascular adipose tissue-derived mesenchymal stem cell; SC-ADSC, subcutaneous adipose tissue-derived mesenchymal stem cell; and SMC, smooth muscle cell.

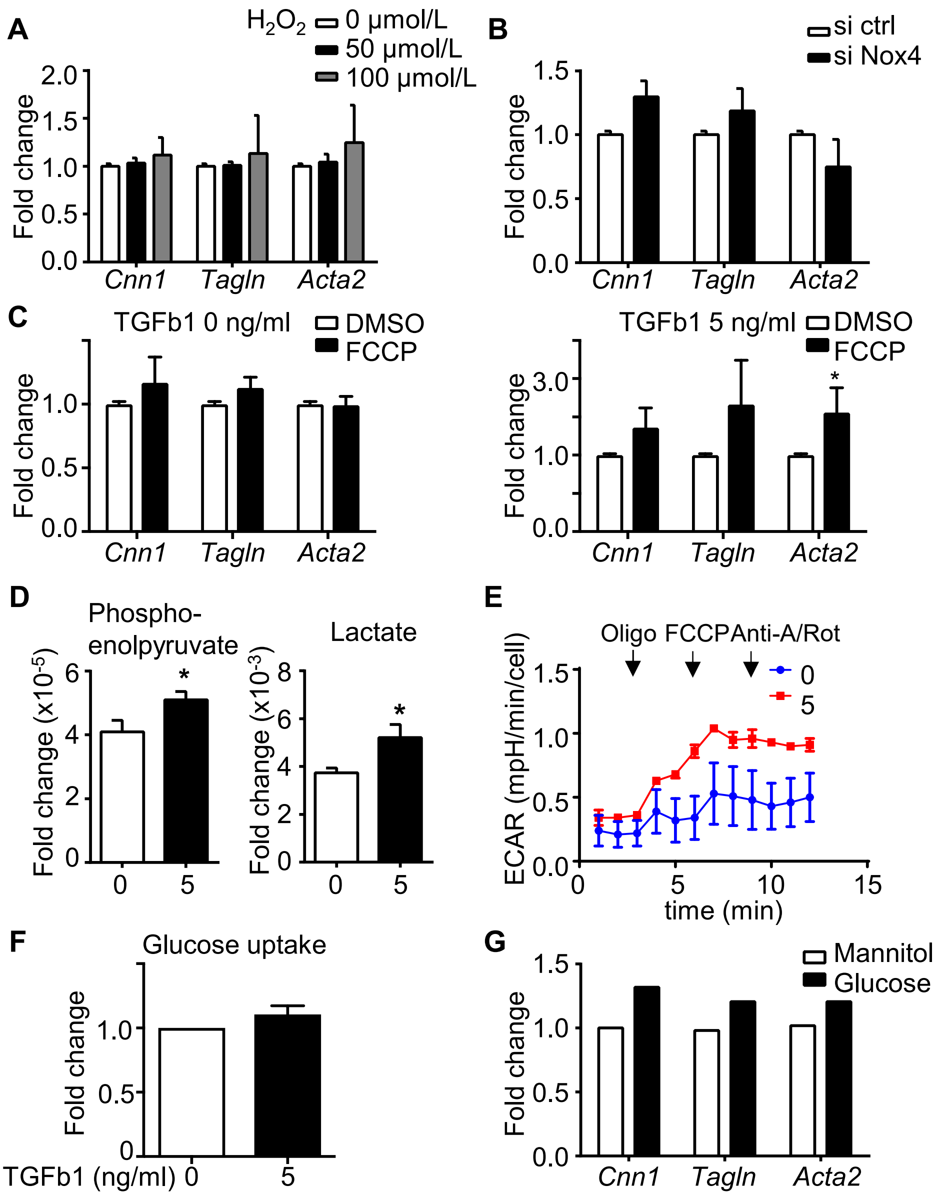

Supplemental Figure XIII. Oxidative stress and glucose level do not influence SMC differentiation. **A**, Cultured PV-ADSCs on the 2nd day of cell seeding were subjected to H_2O_2 treatment for two days (n=3). Data are presented as mean±SD. **B**, Cultured PV-ADSCs on the 2nd day of cell seeding were transfected with 12.5 nmol/L Nox4 siRNA for two days (n=2). Data are presented as mean±SD. **C,** Treatment of cultured PV-ADSCs with (concentration) FCCP for 2 days with or without TGFb1 did not show potent effect in altering SMC marker (*Cnn1, Tagln* and *Acta2*) expression (n=3). DMSO, dimethyl sulfoxide; FCCP, carbonyl cyanide-4-phenylhydrazone. Data are presented as mean±SD. *P<0.05. **D**, Phosphoenolpyruvate and Lactate level after treatment of ADSCs for 4 days with 5 ng/ml TGFb1 (n=3). Data are presented as mean±SD. *P<0.05. **E**, The ECAR rate at the basal level and after the injection of oligomycin, FCCP, anti-mycin A and rotenone was measured in PV-ADSCs cultured with or without TGFb1 for 1 day. ECAR, extracellular acidification rate; Oligo, oligomycin; Anti-A/Rot, anti-mycin A/Rotenone; FCCP, carbonyl cyanide-4-phenylhydrazone. **F**, Glucose uptake was measured in PV-ADSCs treated with 5 ng/ml TGFb1 for 2 days and did not show much change in comparison with cells without TGFb1 treatment (n=3). **G**, Treatment of 25 mmol/L glucose for two days did not induce significant change of SMC markers at mRNA level in PV-ADSCs (n=3).

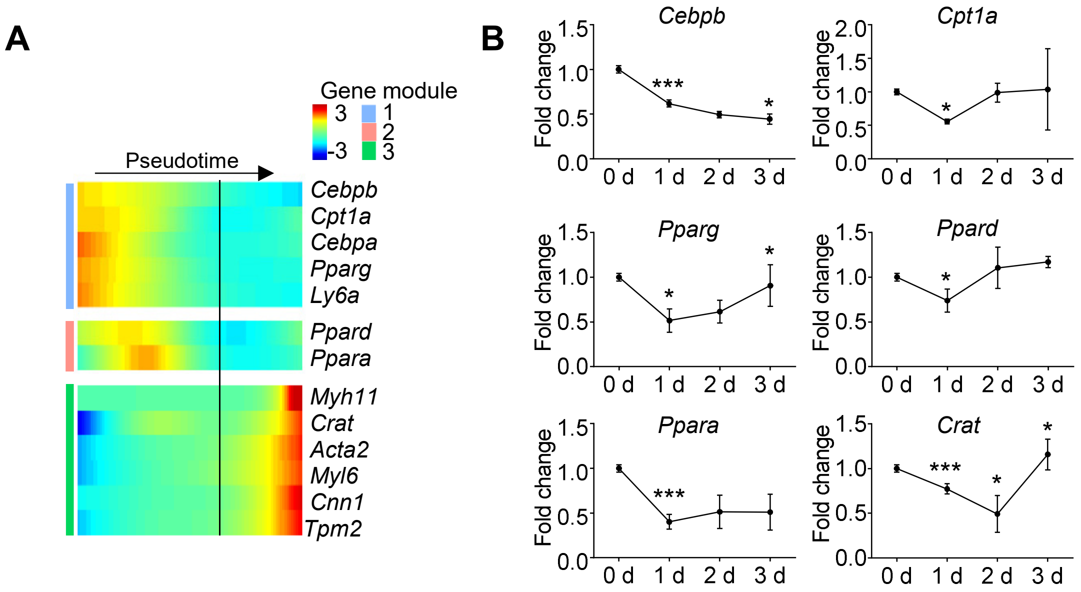

Supplemental Figure XIV. Pseudotime heatmap and real-time change during SMC differentiation from PV-ADSCs. A, SMC markers (*Acta2, Cnn1, Tpm2, Myl6* and *Myh11*) and significantly changed genes related to lipid metabolism (such as *Crat, Cpt1a* and *Cebpb*) along pseudotime were shown as heatmap. Vertical line indicated the downregulation of *Crat* and subsequent upregulation. Color scale, log expression of gene. **B,** Expression of lipid metabolism-related markers at indicated time points (n=3). Data are presented as mean±SD. *P<0.05 and ***P<0.001.

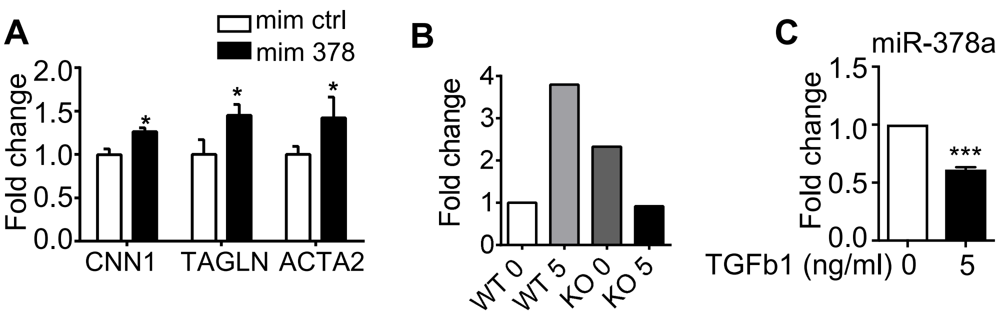

Supplemental Figure XV. Statistic analysis of immunofluorescent staining and effect of TGFb1 on miR-378a. A, Statistic analysis of Figure 7F (n=3). **B**, Statistic analysis of Figure 7I (n=1). **C**, The level of miR-378a-3p was detected with Taqman miRNA assay 2 days after TGFb1 treatment (n=3). Data are mean±SD. **P*<0.05 and ****P*<0.001.

Supplemental Tables

Supplemental Table I. Genes for heatmaps in the Figures.

Fig. 2D Gene name	Fig. 3G Gene module	Fig. 3G TGF-b signaling	Pearson correlation with pseudotime	Fig. 3I Gene module	Fig 3I Transcription factors	Pearson correlation with pseudotime
Col3a1	1	*Ppp2ca*	0.005174	1	***Hif1a***	**-0.13233**
Anxa1	1	*Sp1*	-0.02641	1	***Ebf1***	**-0.24368**
Dcn	1	*Id4*	-0.03443	1	***Stat3***	**-0.11045**
Gsn	1	*Crebbp*	-0.01367	1	***Ebf2***	**-0.13994**
Vim	1	*Inhbb*	-0.04872	1	*Maged1*	-0.08167
Mfap5	1	*Bmpr2*	-0.02602	1	***Zfp36l1***	**-0.11424**
Timp2	1	*Id3*	0.012116	1	***Tcea3***	**-0.08979**
Lum	1	*Lefty1*	-0.01527	1	***Pdlim4***	**-0.09506**
Dpt	1	*Thbs2*	-0.08834	1	***Hbp1***	**-0.11466**
Serpinf1	1	*Acvrl1*	-0.01985	1	***Egr1***	**-0.12748**
C3	1	*Comp*	-0.01303	1	***Uhrf2***	**-0.23956**
Mt1	1	*Tgfbr2*	-0.04926	1	***Bach1***	**-0.11414**
Lgals1	1	*Smad5*	-0.04324	1	***Hoxa5***	**-0.07031**
Rarres2	1	***Dcn***	**-0.34726**	1	***Maf***	**-0.20347**
Rnase4	1	***Tgfbr1***	**-0.15199**	1	***Nfia***	**-0.22692**
Igfbp6	1	*Thbs3*	-0.03599	1	***Thra***	**-0.13762**
Crip1	1	*Rbl2*	-0.04151	1	***Ssbp4***	**-0.10158**
Hsp90b1	1	*Bmpr1a*	-0.03702	1	***Smarca2***	**-0.12274**
Cyb5a	1	*Smad6*	-0.04863	1	***Cebpb***	**-0.30776**
Fosb	1	*Smad3*	-0.05418	1	***Nfe2l2***	**-0.10199**
Gfpt2	2	*Tgfb1*	0.029852	1	***Vdr***	**-0.12711**
Fn1	2	***Ltbp1***	**0.318025**	1	***Wt1***	**-0.16353**
Loxl1	2	***Id1***	**0.203411**	1	***Runx1***	**-0.19621**
Col6a2	2	*Rbx1*	0.094904	1	***Gsc***	**-0.11679**
Nid1	2	*Id2*	0.089395	1	***Aebp1***	**-0.19776**
Fbln2	2	*Mapk3*	0.082398	1	***Nrip1***	**-0.14556**
Scara5	3	*Myc*	0.028472	1	***Cebpa***	**-0.19366**
Pcolce2	3	*Bmp2*	0.047875	1	***Lhx8***	**-0.16394**
Tgfbr2	3	***Rock2***	**0.159384**	2	***Barx1***	**0.109985**
Cd63	3	*Rock1*	0.098576	2	***Hmgb2***	**0.106009**
Cav1	3	*Smad4*	0.036309	2	***Dek***	**0.132678**
Fabp4	3	***Rhoa***	**0.114522**	2	***Nfib***	**0.133017**
Cd36	3	***Tgfb2***	**0.259595**	2	***Ptma***	**0.125247**
Gpihbp1	3	***Inhba***	**0.370686**	2	***Prrx2***	**0.172325**
Srgn	3	*Rps6kb2*	0.038422	2	***Ezh2***	**0.112973**
Tspan13	3	*E2f4*	0.068805	2	***Mef2c***	**0.146496**
Tspan7	3	***Fst***	**0.16016**	2	***Snrpb***	**0.154953**
Cxcl9	3	*Ppp2cb*	0.085641	2	***Uhrf1***	**0.099678**
Adgrl4	3	*Acvr2b*	0.023093	2	***Tmpo***	**0.151375**
Pecam1	3	*Cdkn2b*	0.053425	2	***Tulp4***	**0.134718**
Cdh5	3	*Nog*	0.036384	2	***Lbh***	**0.308562**
Egfl7	3	*Bmpr1b*	0.085947	2	***Nr2f2***	**0.199514**
Cldn5	3	***Tgfb3***	**0.29864**	2	***Sap18***	**0.13799**
Tcf15	3	*Smad7*	0.047004	2	***Hmga2***	**0.129572**

Adgrf5	3	*Smad2*	0.04927	2	***Mllt3***	**0.254327**
	3	*Rbl1*	0.030784	2	***Foxp1***	**0.133072**
	3	*Bmp4*	0.127733	3	***Fhl2***	**0.178154**
	3	*Thbs1*	0.371407	3	***Ankrd1***	**0.25725**
	3	*E2f5*	0.06397	3	***Sertad2***	**0.132396**
				3	***Hes1***	**0.164162**
				3	***Pdlim1***	**0.114697**
				3	***Mtpn***	**0.240424**
				3	***Nupr1***	**0.116644**
				3	***Arid5b***	**0.133602**
				3	***Klf7***	**0.132912**
				3	***Cited2***	**0.175223**
				3	***Basp1***	**0.160311**
				3	***Tgfb1i1***	**0.146746**
				3	***Mkl1***	**0.120295**

Supplemental Table II. GO terms and signalling pathways in Figure 2E and 2F.

Cl 1 GOMF	Cl 1 KEGG	Cl 2 GOMF	Cl 2 KEGG
GO:0005515~protein binding	mmu04514:Cell adhesion molecules (CAMs)	GO:0008201~heparin binding	mmu04512:ECM-receptor interaction
GO:0008289~lipid binding	mmu04670:Leukocyte transendothelial migration	GO:0005201~extracellular matrix structural constituent	mmu04974:Protein digestion and absorption
GO:0005525~GTP binding	mmu04976:Bile secretion	GO:0005518~collagen binding	mmu04151:PI3K-Akt signalling pathway
GO:0071813~lipoprotein particle binding	mmu03320:PPAR signalling pathway	GO:0008083~growth factor activity	mmu04610:Complement and coagulation cascades
GO:0008559~xenobiotic-transporting ATPase activity	mmu05202:Transcriptional misregulation in cancer	GO:0005509~calcium ion binding	mmu05146:Amoebiasis
GO:0008035~high-density lipoprotein particle binding	mmu05160:Hepatitis C	GO:0001968~fibronectin binding	mmu00480:Glutathione metabolism
GO:0005021~vascular endothelial growth factor-activated receptor activity	mmu04510:Focal adhesion	GO:0004364~glutathione transferase activity	mmu04510:Focal adhesion
GO:0042802~identical protein binding	mmu04015:Rap1 signalling pathway	GO:0004602~glutathione peroxidase activity	mmu04141:Protein processing in endoplasmic reticulum
GO:0019903~protein phosphatase binding	mmu04620:Toll-like receptor signalling pathway	GO:0005539~glycosaminoglycan binding	mmu00980:Metabolism of xenobiotics by cytochrome P450
GO:0030169~low-density lipoprotein particle binding	mmu02010:ABC transporters	GO:0005125~cytokine activity	mmu05144:Malaria
GO:0003924~GTPase activity	mmu05200:Pathways in cancer	GO:0050840~extracellular matrix binding	mmu05204:Chemical carcinogenesis
GO:0098641~cadherin binding involved in cell-cell adhesion		GO:0005178~integrin binding	mmu00982:Drug metabolism - cytochrome P450
GO:0043621~protein self-association		GO:0004252~serine-type endopeptidase activity	mmu05133:Pertussis
GO:0030165~PDZ domain binding		GO:0016504~peptidase activator activity	mmu04060:Cytokine-cytokine receptor interaction
GO:0046983~protein dimerization activity		GO:0048407~platelet-derived growth factor binding	mmu05323:Rheumatoid arthritis
GO:0051015~actin filament binding		GO:0016491~oxidoreductase activity	mmu04640:Hematopoietic cell lineage
GO:0008234~cysteine-type peptidase activity		GO:0043295~glutathione binding	mmu04350:TGF-beta signalling pathway
GO:0008013~beta-catenin binding		GO:0008009~chemokine activity	mmu00190:Oxidative phosphorylation
GO:0035091~phosphatidylinositol binding		GO:0008233~peptidase activity	mmu05205:Proteoglycans in cancer
GO:0005215~transporter activity		GO:0005520~insulin-like growth factor binding	

GOMF, gene ontology molecular function

Supplemental Table III. GO terms in Figure 3F

Gene module 1	Gene module 2	Gene module 3
GO:0008201~heparin binding	GO:0003779~actin binding	GO:0044822~poly(A) RNA binding
GO:0005201~extracellular matrix structural constituent	GO:0098641~cadherin binding involved in cell-cell adhesion	GO:0042826~histone deacetylase binding
GO:0005515~protein binding	GO:0051015~actin filament binding	GO:0003723~RNA binding
GO:0048407~platelet-derived growth factor binding	GO:0005515~protein binding	GO:0048306~calcium-dependent protein binding
GO:0005102~receptor binding	GO:0005200~structural constituent of cytoskeleton	GO:0016538~cyclin-dependent protein serine/threonine kinase regulator activity
GO:0005518~collagen binding	GO:0005178~integrin binding	GO:0019901~protein kinase binding
GO:0005178~integrin binding	GO:0005520~insulin-like growth factor binding	GO:0005200~structural constituent of cytoskeleton
GO:0008009~chemokine activity	GO:0019901~protein kinase binding	GO:0098641~cadherin binding involved in cell-cell adhesion
GO:0016504~peptidase activator activity	GO:0019904~protein domain specific binding	GO:0005515~protein binding
GO:0005520~insulin-like growth factor binding	GO:0042803~protein homodimerization activity	GO:0003924~GTPase activity
GO:0050840~extracellular matrix binding	GO:0008083~growth factor activity	GO:0003725~double-stranded RNA binding
GO:0043394~proteoglycan binding	GO:0042802~identical protein binding	GO:0003697~single-stranded DNA binding
GO:0005125~cytokine activity	GO:0017048~Rho GTPase binding	GO:0005525~GTP binding
GO:0016491~oxidoreductase activity	GO:0050998~nitric-oxide synthase binding	GO:0019899~enzyme binding
GO:0003735~structural constituent of ribosome	GO:0005080~protein kinase C binding	GO:0008301~DNA binding, bending
GO:0005507~copper ion binding	GO:0005547~phosphatidylinositol-3,4,5-trisphosphate binding	GO:0042803~protein homodimerization activity
GO:0019838~growth factor binding	GO:0032403~protein complex binding	GO:0008022~protein C-terminus binding
GO:0032403~protein complex binding	GO:0017166~vinculin binding	GO:0003682~chromatin binding
GO:0008237~metallopeptidase activity	GO:0005160~transforming growth factor beta receptor binding	GO:0042393~histone binding
GO:0030984~kininogen binding	GO:0030898~actin-dependent ATPase activity	GO:0061575~cyclin-dependent protein serine/threonine kinase activator activity
